# Land use drives drug resistance in an airborne human fungal pathogen

**DOI:** 10.1093/ismejo/wraf246

**Published:** 2025-11-06

**Authors:** Hylke H Kortenbosch, Bo Briggeman, Francisca Reyes Marquez, Ben Auxier, Sytze de Bruin, Bas J Zwaan, Eveline Snelders

**Affiliations:** Laboratory of Genetics, Wageningen University & Research, Wageningen 6700 AA, Gelderland, The Netherlands; Laboratory of Genetics, Wageningen University & Research, Wageningen 6700 AA, Gelderland, The Netherlands; Centre for Infectious Disease Control, National Institute of Public Health and Environment, Bilthoven 3721 MA, Utrecht, The Netherlands; Laboratory of Genetics, Wageningen University & Research, Wageningen 6700 AA, Gelderland, The Netherlands; Laboratory of Genetics, Wageningen University & Research, Wageningen 6700 AA, Gelderland, The Netherlands; Laboratory of Geo-information Science and Remote Sensing, Wageningen University & Research, Gelderland Wageningen 6700 AA, The Netherlands; Laboratory of Genetics, Wageningen University & Research, Wageningen 6700 AA, Gelderland, The Netherlands; Laboratory of Genetics, Wageningen University & Research, Wageningen 6700 AA, Gelderland, The Netherlands

**Keywords:** *Aspergillus fumigatus*, triazoles, AMR, one health, air sampling, exposure, citizen science

## Abstract

Humans are exposed to the mold *Aspergillus fumigatus* via inhalation, and infections are increasingly resistant to triazole-class antifungals. Ecologically, this fungus is a ubiquitous saprotroph found in terrestrial environments. Although triazole-resistant *A. fumigatus* is found in large quantities in specific agricultural environments, it is not clear how much these contribute to the overall exposure of individuals to antifungal resistance. Triazoles are also used to protect a wide range of products unrelated to agriculture, and therefore, it could not be excluded that the resistance observed in agricultural settings may be the result of selection beyond agricultural sources. In the case of *A. fumigatus,* genomics cannot reliably link resistant isolates to specific environmental sources. Therefore, we used a spatial sampling approach to measure population trends in triazole resistance. We conducted a large-scale, unbiased air sampling throughout the Netherlands using a citizen science approach. We find that $\sim $4$\%$ of over 60K screened colonies are resistant to clinical triazoles. Modeling resistance data with spatial land-use data shows that agricultural land use, particularly flower bulbs and greenhouses, can predict peaks in antifungal resistance in airborne *A. fumigatus* in the Netherlands. Furthermore, genotyping resistant isolates suggests land-use-associated niche differentiation between two dominant resistance haplotypes, with only one of the two showing a significant association with agricultural land use. By linking triazole resistance to land use, this work informs necessary policy-driven changes to reduce human exposure to antifungal-resistant *A. fumigatus*, and suggests that similar spatial patterns in antifungal resistance may occur in other agriculture-associated fungi as well.

## Introduction

The recent COVID-19 pandemic has shown that understanding the transmission of a pathogen is essential to manage the health risks it poses [1], and for airborne pathogenic fungi, we currently lack such understanding. The mold *Aspergillus fumigatus* is an opportunistic human fungal pathogen [2, 3] that causes serious infections in a growing group of patients, often immunocompromized, or suffering from acute or chronic lung disease [4–7]. The increasing frequency of antifungal resistance of *A. fumigatus* to triazole antifungals is of great concern, given both the 25$\%$ increased mortality rate of triazole-resistant infections and the limited alternative classes of antifungals with comparable efficacy [8, 9]. Since it was first proposed in 2009 [10], it has now become established that the use of agricultural triazoles to protect crops against pathogenic plant fungi has led to the selection of cross-resistance to the chemically similar clinical triazoles in *A. fumigatus* in the environment [11–14]. Clinical triazoles such as itraconazole and voriconazole are exclusively used in medicine and not in the environment or agriculture. Resistance has been selected for in the environment by one or multiple agricultural triazoles, the so-called environmental route of clinical resistance, and this has led to cross-resistance to medical triazoles, without actual exposure to medical triazoles in the environment [11, 15, 16].

Although inhalation of airborne spores is considered the primary means of infection with the mold [3, 9], our current understanding of the airborne transmission of (triazole-resistant) spores is poor. In *A. fumigatus*, triazole resistance is most commonly associated with non-synonymous mutations L98H or Y121F/T289A in the *cyp51A* gene, that affect protein folding, together with a repeat of 34 or 46 base pairs in the promotor of the same gene, which increases RNA expression by 1,5 to 2-fold [17, 18]. Mutant analysis has shown that the non-synonymous mutations are not sufficient on their own to confer a high azole resistance phenotype [17, 18]. Similarly, TR duplications alone do not fully account for the resistant phenotype; however, it is the combination that leads to high resistance, which may explain the dominance of the two specific haplotypes, TR$_{34}$L98H and TR$_{46}$Y121F/T289A. Although most of the global *A. fumigatus* population is highly diverse [19], genetic clones can also be detected across the globe [20]. This frequent clonality limits prospects of tracing single or small groups of resistant isolates back to their local source via genomics. Therefore, to systematically identify environmental sources of triazole resistance, a different approach is needed that focuses on spatial population trends instead.

Ecologically, *A. fumigatus* is a ubiquitous saprotroph found in terrestrial environments [3]. Although this fungus is ubiquitous, that does not necessarily imply a uniform abundance [14]. *A. fumigatus* occurs at much higher densities (>10$^{6}$ colony forming units (CFUs)/g) under the nutrient-rich thermogenic conditions found in plant waste heaps prior to composting [13, 21–24], than in top soils (0–10 CFU/g) [25–27]. Flower bulb waste heaps are the most well-studied sources of triazole resistance in the Netherlands, sometimes referred to as resistance hotspots [28], with resistance fractions that readily exceed 50$\%$ [13, 23]. In these Dutch flower bulb waste heaps, TR$_{46}$ haplotypes dominate [23], whereas TR$_{34}$ haplotypes are dominant among clinical isolates and are more commonly observed worldwide [25, 28–33]. Despite anecdotal evidence of the agricultural sources of triazole-resistant *A. fumigatus*, we currently lack comprehensive data on how land use affects its spatial distribution in the air. Temporal studies have suggested that weather effects, such as temperature, precipitation, and wind speed, can affect the airborne abundance of *A. fumigatus* spores [34–36]. Furthermore, industrial-scale composting activity has been shown to increase airborne *A. fumigatus* spores over distances up to 2 km from a source in the United Kingdom [37]. For smaller plant waste heaps, this effect is likely proportionally smaller. However, it is unclear on what scale any composting activity of plant waste piles is associated with which type of land use and how this may spatially affect airborne triazole resistance.

We hypothesize that local agro-industrial activity can have a significant impact on the ecology of *A. fumigatus* populations, particularly levels of antifungal resistance. We build upon a previous publication [12], which used simple passive air samplers in combination with a citizen science approach. This air sampling approach proved effective for collecting airborne *A. fumigatus* and assessing tebuconazole resistance across the UK. In our study, we combine a Citizen Science approach with an improved version of the aforementioned passive air sampling method, validated in a recent methods paper [38]. The new method allows us to phenotypically screen for resistance to two clinically relevant triazoles, itraconazole and voriconazole, at high (>25 CFU) per-sample sampling depth across the Netherlands, providing accurate point estimations of the proportion of resistant spores per sample. We then determine the *cyp51A* promoter haplotypes for the resistant isolates. By combining these data with high-resolution spatial data for land use and weather conditions, we model how these factors contribute to airborne *A. fumigatus* abundance, phenotypic antifungal resistance to itraconazole and voriconazole, and the two dominant resistance haplotypes. In a final set of models we also investigate the impact of fungicide use on airborne *A. fumigatus*.

## Materials and methods

### Recruitment of citizen scientists

The majority of media exposure to the *Schimmelradar* citizen science project was realized through a Wageningen University and Research press release on 6 September 2023 (Supplementary Text A). The press release provided background information on triazole-resistance in *A. fumigatus*, what the objectives of the project were, and a call to action for prospective participants. The release was initially published in the repository of the *Algemeen Nederlands Persbureau* (The General Dutch Press Platform). This resulted in exposure through multiple articles in local newspapers and a national science radio show. In addition, interviews were conducted on local and national radio and television. The recruitment through the media was supplemented with the recruitment through the personal networks of the authors and an advertisement in the Dutch Citizen Science platform newsletter *samenmeten.nl*. During the later stages of recruitment, sparsely populated regions with few registrations were specifically targeted through recruitment on X (https://www.twitter.com">www.twitter.com). Prospective participants were directed to our website (https://www.schimmelradar.nl">www.schimmelradar.nl) through all channels. Here, background information about the project and a sign-up sheet were provided, including information on privacy and data protection regulations.

### Selection of participants

When selecting our participants, we used the R package spcosa [39] to divide the Netherlands into 100 areas of equivalent size (Fig. S1). This division was done naively, without considering local differences in land use. Where possible, we selected an equal number of five participants per area to enhance the spatial coverage of our data and prevent sampling bias. Upon registration, we converted the addresses of the prospecting participants into GPS coordinates. We binned these coordinates into the 100 areas by plotting the points on top of these areas in QGIS (version 3.28.3). We prioritized the first five registrations per area during selection, except when these were spatially clustered and alternative participants were available. If we had fewer than five registrations for an area, additional participants were selected in other areas to fill the spatial gaps there. We rounded the coordinates of the selected participants down to two decimals (the equivalent of a 1 km$^{2}$ area), so they could not be linked to a specific address. We used these rounded coordinates for our spatial analysis and included them only in the data published in conjunction with this manuscript to protect the privacy of our participants.

We gave all selected participants an arbitrary and anonymous sample identifier, independent of location. We then sent all participants an air sampling package, containing a single spore trap as described in the delta trap air sampling protocol included in our 2024 methods paper [38]. The package included all the components required for sampling, written instructions including links to video instructions, and a return envelope (162 mm $\times $ 229 mm) to return the air sample by post free of charge. For an English translation of the instructions given to the participants, including a complete table of contents of the packages, see Supplementary Text B. Participants were instructed to sample outdoor air for four weeks within the window of 1 October to 12 November 2023 at their registered address. We chose this sampling window because the four Dutch samples in our previous pilot study [38] captured sufficient CFUs to reliably quantify triazole resistance at that time of year in the Netherlands. We asked participants to record the start and end dates of their air sampling.

### Air sample processing

Each citizen science sample consisted of three adhesive seals. One seal was used to detect itraconazole resistance, another for voriconazole resistance, and the last seal was used as an extra growth control. We processed air samples with the resistance screening assay validated in our previous methods paper to approximate clinical breakpoints for resistance [38].The Kortenbosch *et al.* [38] assay is a layered culture approach in which adhesive seals are covered with two layers of *A. fumigatus*- selective agar. In the bottom permissive layer, all *A. fumigatus* CFU totals are counted. The upper layer, containing either itraconazole (4 mg/l) or voriconazole (4 mg/l), can only be breached by itraconazole or voriconazole-resistant *A. fumigatus* CFUs, respectively. Counting CFU totals and resistant CFUs from the same sticky seal to calculate resistance fraction allows for more accurate point estimations than relying on separate control and triazole treatment plates. For our CFU counts analysis, we used the median total CFU value out of the three seals in one trap as the response variable. Per area, samples were given priority for processing by order of their date of return and by the best participant compliance with the exposure time and the sampling window. In addition, the samples were processed according to the order of their sample identifier. The air samples were cultured, and resistance fractions were determined within 4 months of taking the sample.

### Genotyping resistant isolates

We genotyped the resistant isolates by first performing a PCR amplification of the *cyp51A* gene, followed by a size differentiation of the amplicons on an agarose gel. First, all *A. fumigatus* colonies from the triazole-supplemented plates were sub-cultured in 96-wells (2 ml, pyramid bottom) (Greiner Bio-One, Kremsmünster, Austria) Malt extract agar (MEA) (1.5$\%$ (w/v) agar) 600 $\mu $l mini-slants [40]. The mini-slants were inoculated with a tungsten needle, from each CFU from the triazole-supplemented plates followed by incubation (2 days, 37$^{\circ }$C). The spores were harvested by pipetting up and down with 600 $\mu $l PBS-0.05$\%$ (v/v) Tween-80 (pH = 7.4). When *Mucorales* spp. were observed near an *A. fumigatus* colony on the original triazole-supplemented plate, the colony of *A. fumigatus* was first transferred to a 60 mm Petri dish with MEA. This subculture was incubated for 2 days at 37$^{\circ }$C and checked for purity before being included in the 96-well slants. Thirty $\mu $l of spore suspension was transferred to a 96-well PCR plate and genomic DNA was isolated from the spores by using a thermal-shock method [40, 41]. In brief, spore suspensions were heated at 95$^{\circ }$C for 15 min in a T100 thermocycler (Bio-Rad Laboratories Inc, Hercules, CA, US), after which the plate was transferred to a $-20^{\circ }$C freezer for at least 1 h. After thawing, the plate was centrifuged and 3 $\mu $l of the supernatant was used as PCR template. The newly designed forward primer TRC51-F1 (5$^\prime $AATCGCAGCACCACTTCAGA$^\prime $3) and established reverse P450-A1 (5$^\prime $ CCATAGCATCGGCACCAT$^\prime $3) primer [42] were obtained from Biolegio (Nijmegen, NL). Twenty-five $\mu $l PCR reactions were prepared using GoTaq G2 DNA Polymerase kit following the instructions from the manufacturer (Promega, Madison, WI, USA). The T100 thermocycler performed one cycle of 95$^{\circ }$C–2 min, 35 cycles of 95$^{\circ }$C–20 s, 58$^{\circ }$C–20 s, 72$^{\circ }$C–1 min, and one cycle of 72$^{\circ }$C–5 min, after finalizing the cycle, the temperature was brought back to 12$^{\circ }$C. The PCR was also performed on DNA of three strains with known *cyp51A* gene promoter haplotypes: a wildtype *cyp51A* strain with no TR, a TR$_{34}$ strain, and a TR$_{46}$ strain. These served as control size markers during gel electrophoresis. The marker’s size was compared with a 100 bp ladder (Thermo Fisher Scientific, Waltham, MA, US) on a 0.5X TAE (Bio-Rad Laboratories) 1.5$\%$ agarose (Eurogentec, Seraing, BE) gel with 0.5 mg/l Ethidium Bromide (EtBr) to confirm the presence of a PCR product. PCR amplicons from all resistant colonies plus control isolates were digested with BglII, which increases the relative size differences between the PCR products, as it cuts the 5$^\prime $ A—GATCT $^\prime $3, outside the duplicated region of the promotor sequence in all haplotypes. For this, 4 $\mu $l of the PCR amplicon was digested using 1 Unit of BglII (Thermo Fisher Scientific) in a total volume of 10 $\mu $l, during 1 h at 37$^{\circ }$C. For a better resolution of the digested fragments, a 3.5$\%$ Low Melting Agarose PPC (Duchefa Biochemie, Haarlem, NL) in 0.5X TAE was used. 5 $\mu $l of the digested PCR product and 1 $\mu $l of each of the control markers were loaded on the gel. The gels were run for 100 min at 150 Volt and stained in 1 $\mu $g/ml EtBr 0.5X TAE buffer for 1 h before imaging using Gel Doc XR+ (Bio-Rad Laboratories) with Image Lab software (Version 6.0.1). The gels were then annotated (see Fig. S9 for an example) for their respective TR haplotype. After determining the TR haplotypes on agarose gel, a subset of PCR products (TR$_{46}$; $n$ = 94, TR$_{34}$; $n$ = 7, larger than TR$_{46}$; $n$ = 17) were sent for Sanger Sequencing (Eurofins, Eusberg, DE) with the NightXpress Mix2Kit. The repeat sequence and length were identified in Geneious Prime (Java Version 11.0.20.1+1 (64 bit)) by mapping to the reference; *cyp51A* promotor region of the *Af293* (NC${\_}$007197.1, [1778907–1783116]) reference genome. This confirmed that the visual interpretation of gel electrophoresis of the digested PCR products was correct, as well as verifying the length of the longer TR haplotypes (TR$_{46^*3}$; $n$ = 15, TR$_{46^*4}$; $n$ = 2). We did not find any TR$_{53}$ isolates [43] in our samples.

### Parameter selection

For our spatial analysis, we used the *Landgebruik Nederland 2022* (LGN2022) [44] grid file that contains Dutch land-use data for 2022. Our air sampling was done in 2023, and the 2023 data were not available at the time of our analyses. Yet, no major changes in land use are recorded on an annual basis. The LGN2022 grid has a spatial resolution of five meters, each classified as one of 51 land-use classes. These classes represent the main crops, forest, water, nature, and urban classes that are compiled from different sources. One of these underlying sources is the *Basisregistratie gewaspercelen* or BRP [45], a public database in which the Dutch government records every year what crops are grown where. All crop classes were retained for our initial land-use analyses. We excluded the nonspecific *Other crops* class and added the *Onions* class from the BRP 2022 dataset because onions occupy the largest surface area grouped under *Other crops* in LGN22. Different categories of forests, surface waters, and urban fabric were generalized to a single class per type of land cover (Table S3). Most nature classes (including dunes, marshlands, and heather and their respective subclasses) were excluded during model selection. For models with significant positive agricultural effects, we ran a second set of high-fungicide land-use models. Here, the general LGN22 crop classes were split when they encompassed specific crop classes with high (>5 kg/ha) recorded intensities of fungicide use (e.g. *Flower bulbs* was split into *Lilies*, *Tulips*, *et cetera*). We used the most recently available fungicide use data [46] from CBS (The Netherlands Statistics Bureau) to select these crops, and extracted the respective spatial data from the BRP 2022 data set. Crops grown in greenhouses were not separated here because greenhouses occupy a relatively small and strongly spatially clustered surface area in the Netherlands. A summarized version of the fungicide-use data (Table S18) and a list of the the variables used from the BRP dataset (Table S3) are included as supplemental materials.

For modeling the median count of colony forming units (CFUs) per trap, we also included weather variables from the AGERA5 database [47]. Namely, wind speed (m/s) at 10 m height, relative humidity ($\%$) at 2 m height, temperature ($^{\circ }$ K) at 2 m height, and precipitation flux (mm/day). These data were extracted from the database for the period overlapping the Citizen science sampling window (1 October 2023 to 12 November 2023) and averaged over the entire period. The distance to the nearest saltwater (based on LGN2022) was also included as a parameter here to check for a potential effect of the distance to the sea (m) on the median CFU count per trap and to check for interaction effects between distance to the sea and weather variables. For all models, the collinearity of the parameters was checked before model selection. In the case of strong co-linearity (>0.7), variables were merged or dropped depending on known or suspected biological relevance.

### Land and fungicide use data extraction

For each variable included in our models, we generated land-use heat maps. The 5 m resolution pixels were first aggregated into 250-m pixels for computational efficiency. Each larger pixel retained the proportional composition of the smaller pixels in the land-use classes in our models. For example, if 10$\%$ of the 5 m pixels within a 250 m pixel were classified as forest, the forest value for the larger pixel was set to 0.1. Subsequently, we determined the mean value for all land-use variables within a 10 km radius around each 250 m pixel to generate heat maps. We then extracted the land-use proportions from the heat maps at all locations where air samples were taken and linked them to each air sample site for modeling. Spatial distributions of all land-use and weather variables included during model selection can be found in Figs 2 and S4. Given that spores can disperse directly across a few km through the air [37], the 10 km radius was chosen to account for both the known dispersal range of *A. fumigatus* and the fact that where a crop is grown is typically not where its waste is processed. Early on during modeling, a 5 km range was also tested and found not to improve model predictions. To generate the spatial distribution of Dutch fungicide use, we again generated land-use heatmaps. This was done the same as described above, but now for all crops for which we had both publicly available CBS fungicide sales data [46] and spatial distributions from the BRP dataset [45]. We weighted these heatmaps by the reported annual dosage for each respective crop (kg/ha) and summed the weighted heatmaps to generate our spatial estimate of Dutch fungicide use (Fig. S10).

### Data analysis and visualization

All data analysis was done in R (version 4.4.0) [48]. To test for significant differences in the proportion of the TR$_{34}$ and TR$_{46}$ genotypes on our paired itraconazole *versus* voriconazole plates, we used McNemar’s test from the stats R package [48]. We used generalized linear models (GLMs) of the beta-binomial family of the GlmmTMB package [49] to model both the phenotypic resistance and haplotype data. We specified the total CFUs per triazole-treated seal as the number of trials and the number of colonies resistant to itraconazole or voriconazole, or resistant TR$_{34}$ or TR$_{46}$, isolates as the number of successes on each respective seal. Combining the number of trials and successes, we modeled the likelihood of resistance given the number of screened colonies as the response variable in the phenotypic resistance and haplotype models. Weather data from the AgERA5 was loaded into R using the ncdf4 package [50], whereas the R package raster was used to handle spatial data in R [51]. We used a GLM of the negative binomial family from the MASS package [52] to model the median CFU count per sample. CFU counts were normalized for an exposure time of 28 days when there were deviations of 1 or more days from this instructed exposure time. Parameters and respective datasets included during model selection can be found in Tables S3 and S29. All GLMs were two-tailed, and the 355 air samples used in the models were biological replicates. The Akaike information criterion (AIC) was used to select the most informative models. Five-fold cross-validation was used to test whether the selected models provided a meaningful decrease in root mean square error (RMSE) or increased predictive accuracy, relative to the relevant null model. Model averaging was performed on all models within two AIC units of the minimal AIC model using the R package MuMIn [53]. AIC values were used as model weights when computing the weighted average of coefficients across the models. The minimal AIC models were used to generate the spatial predictions using the R package terra [54], whereas the averaged models were used for model interpretation. Variance inflation factors were assessed for all models with the R package performance [55]. The packages sf [56] and stars [57] were used to handle spatial data. Semi-variograms of all spatial models were used to check for spatial autocorrelation in the data. Semi-variograms were generated using the gstat R package [58]. The plots were generated using the ggplot2 package [59] and the map of the Netherlands was generated using the tmap package [60].

## Results

### Sample overview

The successful recruitment for the citizen science project *Schimmelradar* (in English “Fungal radar”) led to 8,605 registrations, from which we selected 503 participants to whom we distributed air sampling packages to maximize spatial coverage (Fig. S1). Of these, 412 (81.9$\%$) were returned. 47 late-arriving samples of good quality were not used in this study because they were taken in a region where we already had sufficient (>3) processed samples. Of the 365 samples we processed, five could not be used for resistance fraction estimations because of too few CFUs <25 [38], and five with >300 CFUs were excluded because of inaccurate counts due to colony overcrowding. Our final sample size was therefore 355 (Table S1). An overview of the citizen science project, including project description and public communications, is found in Supplementary File A. We screened 29 236 *A. fumigatus* colonies for voriconazole resistance, observing an overall resistance of 3.36$\%$, and screened 30 391 colonies for itraconazole resistance, where we found an overall resistance of 4.57$\%$ (Table 1). Underlying the phenotypic triazole resistance fractions, TR$_{34}$ ($n$ = 850) and TR$_{46}$ ($n$ = 808) are the two most dominant resistance haplotypes, making up 90.5$\%$ of phenotypically triazole-resistant isolates (Table S2). TR$_{34}$ was dominant (53.1$\%$) on itraconazole, whereas TR$_{46}$ was dominant (61.4$\%$) on voriconazole selective media (Table S2).

**Table 1:** **Summary statistics of the *A. fumigatus* population**, including sample CFU counts and resistance (%) based on the 355 analyzed *Schimmelradar* samples.

	Population total	Sample mean	$\pm $ SE	Sample median
		($N$ = 355)		($N$ = 355)
Normalized median CFU per trap^a^	29 057	81.9	1.49	79
Voriconazole screened CFU	29 236	82.4	1.71	80
Voriconazole resistant CFU	914	2.57	0.157	2
Voriconazole resistance ($\%$)	-	3.36	0.955	2.22
Itraconazole screened CFU	30 391	85.6	1.64	82
Itraconazole resistant CFU	1346	3.79	0.178	3
Itraconazole resistance ($\%$)	-	4.57	1.11	3.90

$^{\mathrm{a}}$ Because the three seals within a trap are not independent samples, this refers to the seal with the median counts per trap. Counts were normalized by exposure time.

### Local differences in airborne *A. fumigatus* abundance

**Figure 1:**
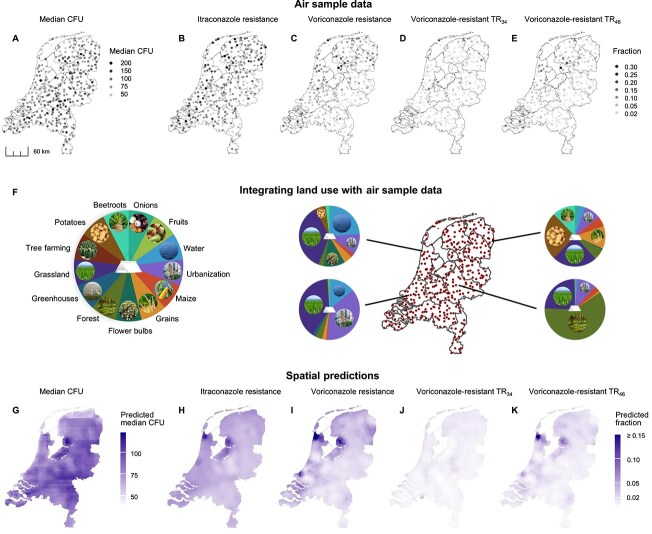
Overview of the raw spatial air sampling data (A–E), integration of air sample and land-use data (F), and spatial predictions based on the land-use models (G–K). For the raw data plots (A–E) each dot represents a sample location and the grayscaling represent the number of captured CFUs (A), the fraction of phenotypical resistant colonies for itraconazole (B) and voriconazole (C), or the fraction of colonies harboring TR$_{34}$ resistance haplotype (D) or the TR$_{46}$ resistance haplotype (E) per voriconazole-screened sample (per itraconazole-screened sample can be found in Fig. S5). A schematic representation of how the proportions of the different land-use classes within a ten km circular radius of each sample were integrated with the air sample data, including four examples highlighting that land use varies across the Netherlands (F). The spatial predictions across the Netherlands are shown based on best-fitting land-use models of the air sample data, and presented in the same order as in the top panel (G–K).

**Figure 2:**
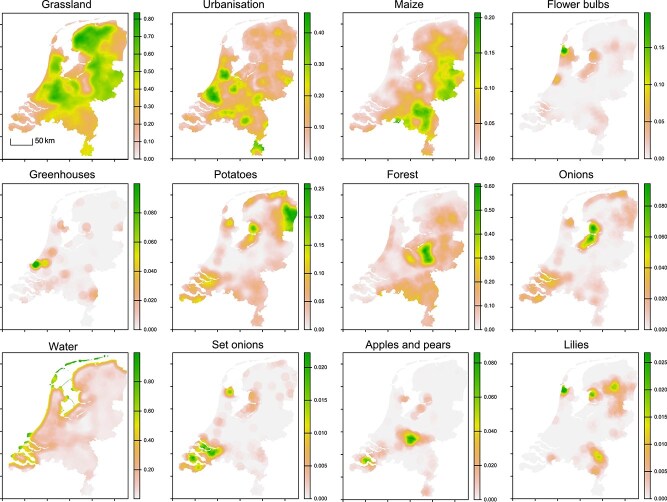
Spatial distributions of the proportion of land-uses within 10 km of any given point within the Netherlands, the land use data were extracted from the LGN2022 [44] and BRP [45] databases. Spatial distributions of the proportion of land use within 10 km of any given point within the Netherlands. These maps were used for the extraction of the proportions of the different land uses around each of the sampling sites, as well as the spatial predictions based on model output. Continued in Fig. S4.

The total CFU counts, indicating airborne *A. fumigatus* abundance, varied by about one order of magnitude (min. 25– max. 300) (Fig. 1A). We found several significant relationships between airborne *A. fumigatus* abundance and spatial variables (Tables S4–S5). CFU counts were lower near coastal areas, with strong negative associations ($\beta $ = −1.23, SE = 0.239, *P* = <.01) with the land cover class *Water* and proximity to salt water (m) ($\beta $ = $1.84 \times 10^{-6}$, SE = $6.62 \times 10^{-7}$, *P* = <.01) (Figs 1A and G). Here ($\beta $) represents the log-expected counts and indicates the direction of the slope of the correlation between the explanatory and response variable (the CFU count). We also observed weaker negative associations with *Forest* and *Grassland*. For an overview of the spatial distributions of Dutch land uses discussed in the main text, see Fig. 2. Climatic variation also explained variation in *A. fumigatus* abundance: higher temperatures and relative humidity were associated with increased CFU counts, whereas precipitation was negatively associated with abundance. For the full model description, see Tables S4 and S5. As we did not *a priori* expect an effect of climate on triazole resistance, we did not model climate data for these response variables.

### Land use is highly associated with triazole resistance

Itraconazole resistance was more prevalent than voriconazole resistance, occurring in 90.7% (322/355) of samples compared to 76.1% (270/355). Resistance to at least one of the two triazoles was detected in 96.3% of samples. Itraconazole resistance ranged from 0% to 21.3% per sample (Fig. 1B), with values above 10% more common in the Northwestern part of the country. Voriconazole resistance showed a more pronounced regional pattern, reaching up to 30.9% in the Northwest, while remaining lower in the Southeast (Fig. 1C).

The TR$_{34}$ haplotype was relatively evenly distributed across the country (Fig. 1D), while TR$_{46}$ occurred in distinct hotspots that overlapped with areas of high voriconazole resistance (Fig. 1E). Resistance to itraconazole and voriconazole was moderately correlated ($R^{2} = 0.39$, Fig. S3A), whereas the TR$_{34}$ and TR$_{46}$ haplotypes were spatially uncorrelated ($R^{2} = 0.03$, Fig. S3B). TR$_{46}$ was more strongly associated with phenotypic voriconazole resistance ($R^{2} = 0.54$, Fig. S3E) than TR$_{34}$ was with itraconazole resistance ($R^{2} = 0.24$, Fig. S3D).

Spatial models revealed distinct land-use predictors for phenotypic resistance (Fig. 1H–I; Tables S6–S9). For itraconazole, no significant positive predictors were identified. However, *Maize* and *Forest* showed significant negative associations with resistance fractions. For the full model output, see Tables S6 and S7.

**Figure 3:**
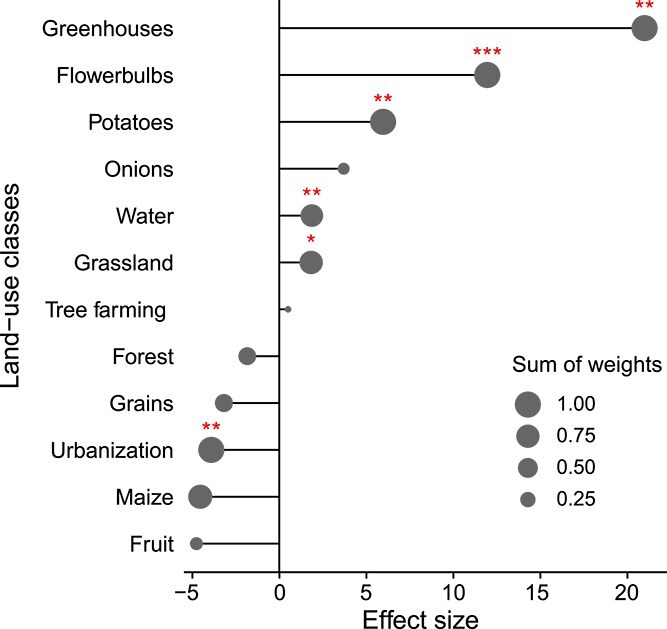
Lollipop plot of the effect of the different land-use classes on phenotypic voriconazole resistance. The X-axis represents the log odds of the effect size ($\beta $), while the size of the heads indicates the Sum of Weights of each class. (^*^ = $P<$.05, ^*^^*^ = $P<$.01, ^*^^*^^*^ = $P<$.001).

In contrast, voriconazole resistance was positively associated with several agricultural land uses (Fig. 3). The strongest predictors were *Greenhouse* ($\beta $ = 20.2, SE = 6.38, *P* <.01), *Flower bulb* ($\beta $ = 11.5, SE = 3.32, *P* <.01), and *Potatoes* ($\beta $ = 5.74, SE = 1.82, *P* <.01). Weaker positive associations were found for *Grassland* and *Water*. Negative predictors included *Urbanization* and *Maize*. For the full model output, see Tables S8 and S9. Here and all following resistance models, ($\beta $) represents the log-odds of the effect size and indicates the direction of the slope of the correlation between the explanatory and response variable (the probability that a CFU is resistant).

**Table 2:** Overview of the model performance of all models discussed in the results section.

**Response variable**	**Parameters**	** $\Delta $ AIC rel.**	**NRMSE**	** $\Delta $ RSME (%)**
		**to null model**		
Itraconazole resistance fraction	Land use (LGN)	**−34.1**	0.168	**5.04**
Voriconazole resistance fraction	Land use (LGN)	**−94.5**	0.114	**9.77**
Voriconazole-resistant TR$_{34}$ fraction	Land use (LGN)	**−14.6**	0.128	3.34
Voriconazole-resistant TR$_{46}$ fraction	Land use (LGN)	**−44.6**	0.0839	**6.50**
Median CFU counts	Land use (LGN) + weather (AgERA5)	**−81.4**	0.125	**8.16**
Itraconazole resistance fraction	LGN + high-fungicide land use (BRP)	**−37.3**	0.166	**6.37**
Voriconazole resistance fraction	LGN + high-fungicide land use (BRP)	**−99.5**	0.108	**13.90**
Voriconazole-resistant TR$_{46}$ fraction	LGN + high-fungicide land use (BRP)	**−52.7**	0.0831	**7.52**
Itraconazole-resistant TR$_{46}$ fraction	LGN + high-fungicide land use (BRP)	**−68.8**	0.105	**8.73**
Itraconazole resistance fraction	Fungicide use	**−13.9**	0.175	1.20
Voriconazole resistance fraction	Fungicide use	**−29.9**	0.120	4.58

The table outlines which response variables were modeled with parameters selected from which datasets. $\Delta $AIC relative to the null indicates improvement to the model fit. The normalized root mean square error (NRSME) is a metric of model accuracy. The $\Delta $RSME indicates model accuracy improvement relative to the null model. We consider a model with a $\Delta $AIC of < $-10$ and $\Delta $RSME of > 5% to be meaningfully more predictive than its respective null model (bold). For a full summary of model performance, see Table S27.

### Distinct spatial distribution of resistance haplotypes

Voriconazole-resistant TR$_{46}$ showed strong spatial overlap with overall phenotypic voriconazole resistance (Fig. 1K), driven by similar land-use predictors. *Flower bulbs* was a significant positive predictor ($\beta $ = 10.4, SE = 4.79, *P* =.030), with *Greenhouses* also showing a positive but non-significant effect ($\beta $ = 17.6, SE = 9.28, *P* =.058). These predictors mirrored those for phenotypic voriconazole resistance (Fig. 1I), consistent with their spatial correlation ($R^{2} = 0.54$, Fig. S3E). Several land-use classes were significantly negatively associated with airborne TR$_{46}$, including *Urbanization*, *Maize*, *Forest*, and *Water*, also overlapping with the phenotypic voriconazole resistance model. For the full model description, see Tables S10 and S11. In contrast, no land-use class had a significant positive effect on voriconazole-resistant TR$_{34}$ (Tables S12 and S13). The model prediction was flat (Fig. 1J), with only *Urbanization* and *Maize* showing significant negative effects. This suggests that whereas TR$_{46}$ is spatially linked to large-scale agriculture, the spatial distribution of TR$_{34}$ is not. A similar haplotype-specific spatial relation to land use was found when focusing on itraconazole-resistant isolates, with only the TR$_{46}$ haplotype showing significant land-use associations (Tables S14–S17).

### Fungicide use and airborne triazole resistance

To assess whether agricultural fungicide application drives airborne resistance to clinical drugs, we tested for a dose-response relationship. We refined broad land-use classes (*Flower bulbs*, *Onions*, *Potatoes*) from LGN [44] into crop-specific categories using the BRP dataset [45], based on their known high fungicide use (Table S18). Including these refined high-fungicide land-use classes during model selection improved model fit and predictive accuracy for both phenotypic resistances and the TR$_{46}$ haplotype (Table 2).

*Lilies* emerged as the most consistent and strongest positive predictor across models. It was significantly associated with phenotypic itraconazole resistance ($\beta $ = 39.7, SE = 16.4, *P* =.016), voriconazole resistance ($\beta $ = 79.4, SE = 22.4, *P* <.01), itraconazole-resistant TR$_{46}$ ($\beta $ = 77.7, SE = 31.5, *P* =.014), and voriconazole-resistant TR$_{46}$ ($\beta $ = 74.6, SE = 34.4, *P* =.030). *Set Onions* was also a strong positive predictor, though only for the voriconazole-resistant TR$_{46}$ model. For the full high-fungicide models, see Tables S19–S26 and Figs S6–S8.

To directly explore the effect of agricultural fungicide use in the absence of fungicide-use data, we created a spatial approximation of fungicide use by integrating crop-level 2020 sales data from the CBS [46] with spatial distributions of crops from the BRP dataset. This agricultural fungicide-use proxy was significantly positively associated with phenotypic voriconazole ($\beta $ = 0.550, SE = 0.0859, *P* <.01) and itraconazole resistance ($\beta $ = 0.303, SE = 0.07, *P* <.01). However, fungicide use alone was a weaker predictor of phenotypic resistance (itraconazole: $\Delta $RMSE = 1.20%, voriconazole: $\Delta $RMSE = 4.48%) relative to the respective fungicide-naive land use models (itraconazole: $\Delta $RSME 5.04$\%$, voriconazole: $\Delta $RSME 9.77$\%$) (Table 2). This indicates that local land use provides additional explanatory power beyond the predicted local fungicide use.

## Discussion

### Antifungal resistance and agriculture

Our strongest model results described the associations between voriconazole resistance and land uses. The two most robust correlations we found between voriconazole resistance and agricultural land uses are *Flower bulbs*, a common target of resistance monitoring in the last decade [23, 28], and *Greenhouses*. To our knowledge, greenhouse plant waste material has not been surveyed in the Netherlands for potential resistance hotspots, although a study in China [61] found that 30$\%$ of *A. fumigatus* in greenhouse topsoil were cross-resistant to itraconazole and voriconazole. Our air sampling data predict that the Dutch greenhouse sector is also associated with sources of triazole resistance. However, given our unbiased sampling approach and the relatively small area used for greenhouses in the Netherlands, more targeted sampling will be needed to pinpoint which greenhouse crops underlie this association. Both the *Flower bulb* and *Greenhouse* land-use classes include crops with the highest annual fungicide use per hectare in the Netherlands [46]. Perhaps unsurprisingly, the crop with the highest overall use of fungicides in the Netherlands; *lilies* (28 kg$\cdot $hectare$^{-1}\cdot $year$^{-1}$, compared to 4.9 kg$\cdot $hectare$^{-1}\cdot $year$^{-1}$ across all crops) [46], is the most robust positive predictor of voriconazole resistance and detection of TR$_{46}$ haplotypes. Due to global variation in land use, fungicide use and waste management, the associations we find between Dutch land-use and triazole resistance associations may not explain global patterns of airborne triazole resistance [31–33, 62–66]. However, we do expect that the general principle of land use driving local airborne triazole resistance in conjunction with fungicide use will be applicable globally.

The study of Shelton *et al.* [12] was the first to carry out environmental air sampling with the help of citizen scientists to screen for (triazole-resistant) airborne *A. fumigatus*, but did not find spatial correlations between (triazole-resistant) airborne *A. fumigatus* and land use. The analysis in this previous work was carried out using presence-absence data rather than quantitative spore counts and resistance proportions, which we were able to include in the work presented here by building upon the earlier method. This study detects spatial associations, primarily due to a greater sampling depth and the inclusion of crop-specific spatial data in a radius around the sampling sites during model selection. This finding represents a key conceptual leap forward in understanding the underlying causes of airborne triazole resistance in *A. fumigatus*.

### Spatial differences among resistance genotypes

In Dutch air, we find a roughly equal overall fraction of TR$_{34}$/TR$_{46}$ haplotypes. This contrasts with Dutch clinical data [29] where TR$_{34}$ was twice (55$\%$) as common as TR$_{46}$ (28$\%$) among Dutch resistant infections in 2023 [67]. This paradox may be explained by the distinct spatial distributions we recovered, which provide comprehensive evidence of niche differentiation between these dominant triazole resistance haplotypes. We found pronounced peaks in the TR$_{46}$ fractions in areas distant from urban centers; in contrast, the TR$_{34}$ haplotype had a more uniformly low (<4$\%$) occurrence. Both the TR$_{46}$ fraction and phenotypic voriconazole resistance were significantly correlated with agriculture, including the described resistance hotspots [23]. This suggests that, at least in the Netherlands, the environmental niche of the TR$_{34}$ haplotype is currently not strictly associated with agricultural activity. Therefore, patients at risk outside of the high TR$_{46}$ regions may have a higher chance of exposure to TR$_{34}$ than TR$_{46}$, which may explain the dominance of TR$_{34}$ in the Dutch clinical data. Identifying the selective environments for these haplotypes is imperative, as their differences in specific antifungal susceptibility patterns may have important implications for the preferred clinical treatment [13, 30, 68].

What could drive these haplotypes to occupy different niches? First, the different distributions could be the result of a later origin of TR$_{46}$. To our knowledge, the first recorded TR$_{34}$ isolate stems from 1998 [2], while the earliest TR$_{46}$ isolate was recorded in 2009 [69]. The TR$_{46}$ haplotype may simply not have had as much time to disperse, but given the global spread of TR$_{46}$ in the last decade [31–33, 62–66] this seems unlikely. Alternatively, natural selection could explain the spatial distribution, with the TR$_{46}$ haplotype being more fit under the multi-fungicide cocktail of agricultural conditions [23, 28], or suffering from a higher fitness cost in environments without fungicide pressure compared to the TR$_{34}$. Although environmental fitness and costs of resistance to fungicides are understudied in *A. fumigatus*, Chen et al. [70] recently demonstrated that the genomic background of TR$_{46}$ isolates, but not the *cyp51A* alleles themselves, more often carried a fitness cost compared to TR$_{34}$ isolates. This fitness cost in the absence of fungicides could restrict the spatial distribution of TR$_{46}$ isolates across the country and therefore limit exposure to patients at risk. A combination of legacy and adaptive factors could also be at play. The TR$_{34}$ haplotype is known to be environmentally selected and not the product of in-host adaptation [19]. Given that TR$_{34}$ has been detected a decade before TR$_{46}$ it may also have had more time to adapt to non-fungicide environments and therefore by 2023 lack a strong correlation to specific land uses.

### Societal implications

We find a mean phenotypic resistance of 3.4$\%$ for voriconazole and 4.5$\%$ for itraconazole in airborne *A. fumigatus* in the Netherlands, and only 13/355 sample locations showed no antifungal resistance for both clinical compounds. These findings highlight both the prevalence of airborne exposure to antifungal resistance and the selection for cross-resistance to these clinical triazoles, which are not applied environmentally themselves [46]. Despite previous knowledge of the general conditions that allow for triazole resistance selection in plant waste heaps [10, 28], it has been unclear how dispersal of spores affects the wider environment. Using land use data, we can predict the variation detected in phenotypic airborne triazole resistance and the TR$_{46}$ haplotype. Therefore, to maximize available resources, future studies to identify resistance hotspots may implement hierarchical sampling with initial low-scale sampling to identify potential regional resistance levels, and reserve subsequent fine-scale sampling for regions with elevated resistance to identify spatial associations in triazole resistance. With a method in hand suitable for large-scale applications, proper integration of spatial metadata around unbiased sampling sites is key to moving beyond incidental sampling. Here, we demonstrate that integration of air sample and land use data can predict agricultural sources of triazole resistance for the TR$_{46}$ haplotype. For the TR$_{34}$ haplotype specifically, the land use data we used for modeling did not appear to contain effective spatial predictors. If discrete sources of the TR$_{34}$ haplotype do exist, both finer-scale air sampling and smaller-scale metadata on local non-agricultural plant waste may be needed to identify those.

Our finding that naïve land-use models outperform fungicide-use-proxy models in predicting phenotypic triazole resistance suggests that fungicide use data is not strictly necessary to identify agricultural resistance hotspots, although such data remain valuable. Several high-fungicide crops were positively associated with airborne resistance and the TR$_{46}$ haplotype, although not all crops showed this pattern. Crops such as apples and pears, despite their high fungicide input, did not show a significant association. Direct sampling data of *A. fumigatus* resistance in fruit waste is sparse, but a study that did sample such waste also found triazoles but no *A. fumigatus* [28]. These observations highlight how fungicide use alone does not fully account for the spatial patterns of airborne resistance. Other agricultural factors likely contribute to resistance selection and amplification [13, 21–23]. For example, crop waste management practices that facilitate the growth and dispersal of *A. fumigatus* [28], such as piling up plant waste material for weeks or months, can allow natural selection to act on antifungal resistance. In contrast, land uses where agro-industrial plant waste heaps containing fungicides are expected to be sparse, such as forest or urbanization, show several significant negative correlations to triazole resistance. The same applies to maize, which is grown at a large scale yet sees no recorded fungicide use in the Netherlands in the most recent reported overview (2020) [46]. Although the conditions that favor resistance selection may differ between fungal species, including fungal pathogens, it is unlikely that the effects of fungicide use are exclusive to the human fungal pathogen *A. fumigatus*. A recent study of the European population of *Blumeria graminis forma specialis tritici* (Wheat powdery mildew) suggests that spatial population trends in antifungal resistance related to local differences in fungicide use may be present in plant pathogenic fungi as well [71]. Plant pathogenic fungi and human pathogenic fungi have shown increasing trends of antifungal resistance, particularly in the past decade [72, 73]. Our work illustrates that land use can serve as a proxy for the many factors and farming practices (e.g. local fungicide use) that can contribute to antifungal resistance selection, for which comprehensive data are often lacking.

Ultimately, triazoles have a societal value in protecting crops against fungal plant pathogens, but this class also has important clinical value as a first-line treatment in the management of fungal diseases. Therefore, dual use of this class creates a conflict due to cross-resistance between agricultural and clinical triazoles. Understanding the role of the various factors underlying the correlation of particular land uses with airborne antifungal resistance is essential to solving this multi-stakeholder problem. Air is the ultimate dispersal mechanism through which humans are exposed to spores, and aerial surveys inform us of the ultimate (resistant) sources of this human fungal pathogen.

## Supplementary Material

ISME_Schimmelradar_sup_fig_tab(4-12-25)_wraf246

ISME_s_text_Schimmelradar_(30-10-25)_wraf246

## Data Availability

Raw data and analysis code used in this study and required to re-analyze the data are publicly available at https://doi.org/10.5281/zenodo.15101989

## Materials availability

*Aspergillus fumigatus* isolates of this study that have been stored will be made available by contacting E.S.
