# Supplementary material for: Land use drives drug resistance in an airborne human fungal pathogen
Source: ISME J. 2025 Nov 6;19(1):wraf246. doi: 10.1093/ismejo/wraf246 (PMC12646274; doi:10.1093/ismejo/wraf246)
Supplement: ISME_Schimmelradar_sup_fig_tab(4-12-25)_wraf246 [file isme_schimmelradar_sup_fig_tab(4-12-25)_wraf246.pdf]

Supplementary Figures and Tables of:

Land use drives drug resistance in an airborne human fungal pathogen

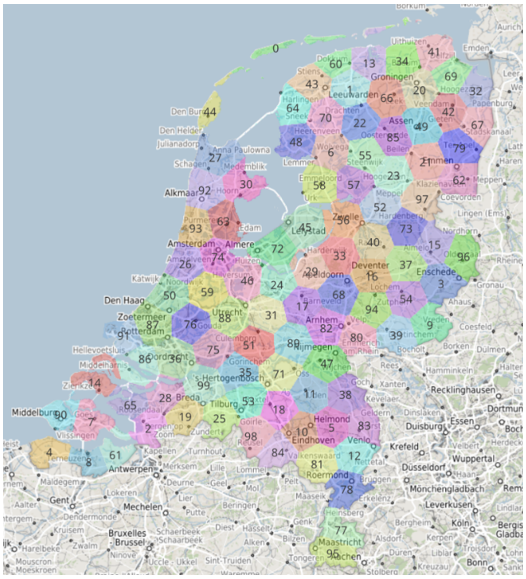

Fig. S1. Subdivision of the Netherlands. The 100 equisized areas in which the Netherlands was divided using the Spcosa R package to facilitate selection of participants across the county to ensure spatial coverage. The numbers are random and have no meaning beyond designating the different areas. Map data from OpenStreetMap `https://www.openstreetmap.org/copyright`.

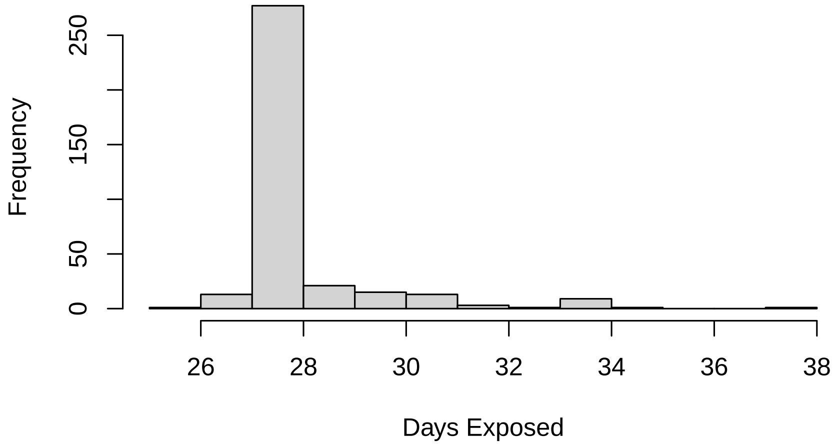

Fig. S2. Histogram of the number of days participants exposed the traps. The majority of participants of the 355 analyzed samples reported compliance to the 28-day exposure time. For the analysis of median CFU per trap the counts of all samples were normalized by exposure time

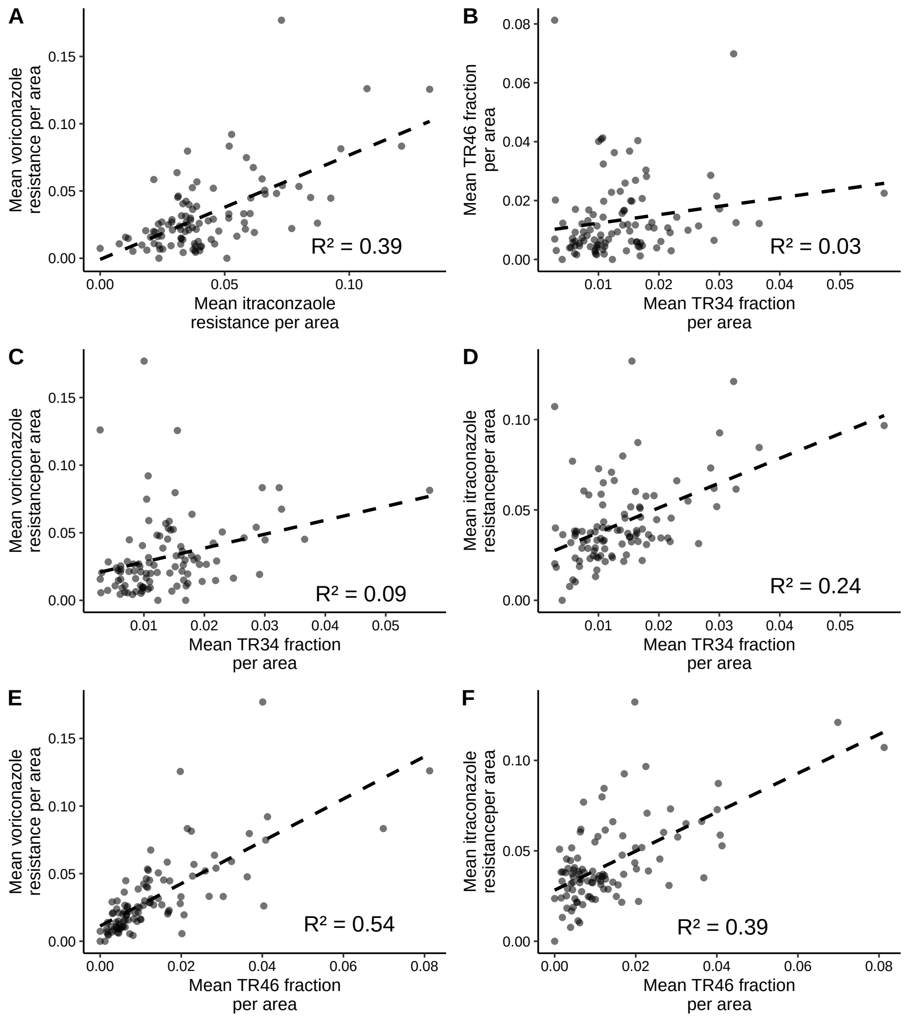

Fig. S3. Correlations between phenotypic resistance and genotypic resistance fractions. Dots represent the fraction of the mean counts of the seals within each of the 100 sampling areas.

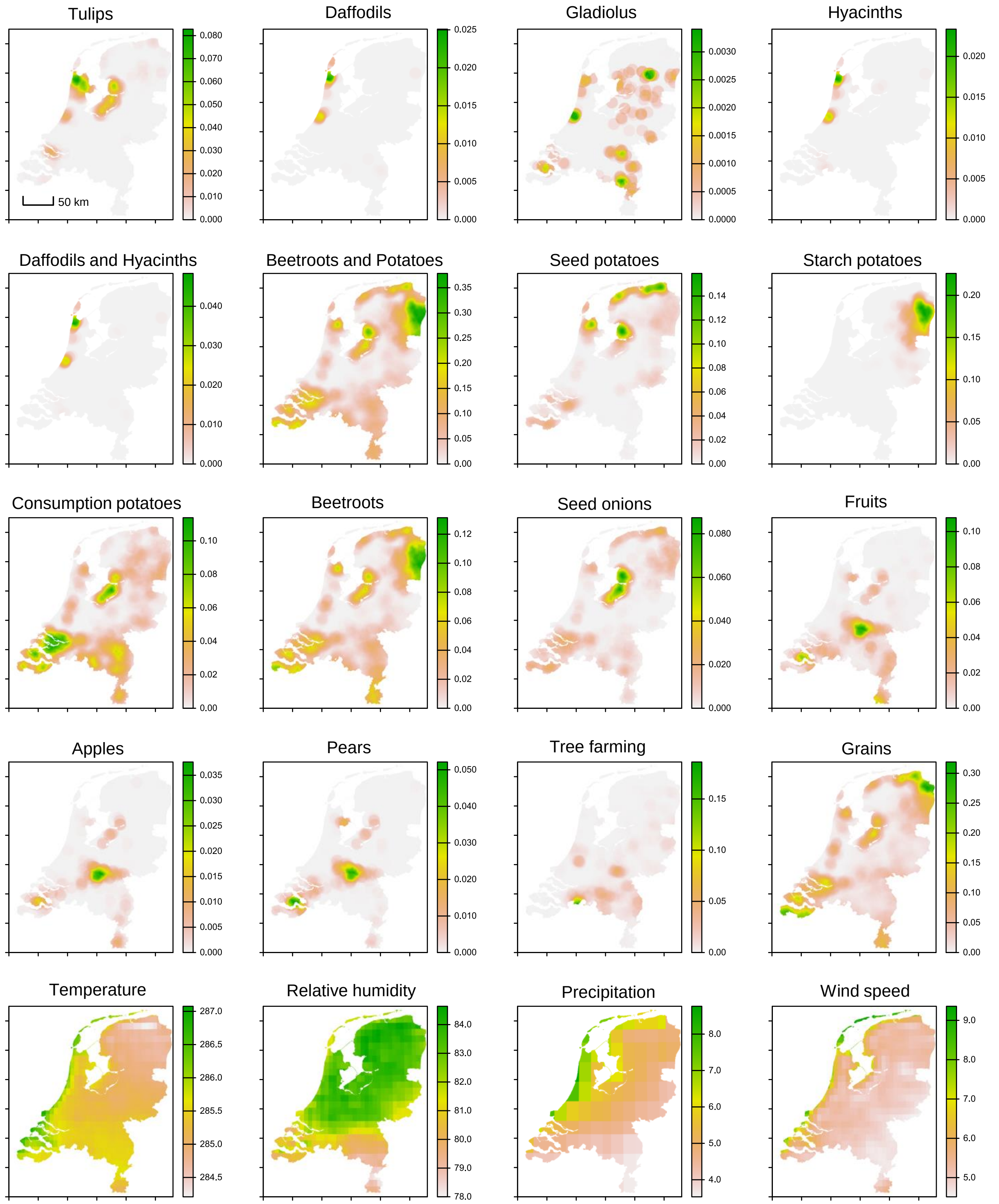

Fig. S4. (Continuation of Figure 2). Spatial distributions of the land-use variables extracted from the LGN2022 and BRP databases. Spatial distributions are of proportion of land use within 10 km of any given point within the Netherlands.
The bottom four panels include spatial weather data extracted from the AgERA5 database for the sampling window set to the citizen scientists. Windspeed is in m/s, temperature in °K, relative humidity in %, precipitation flux in mm day^{-1}.

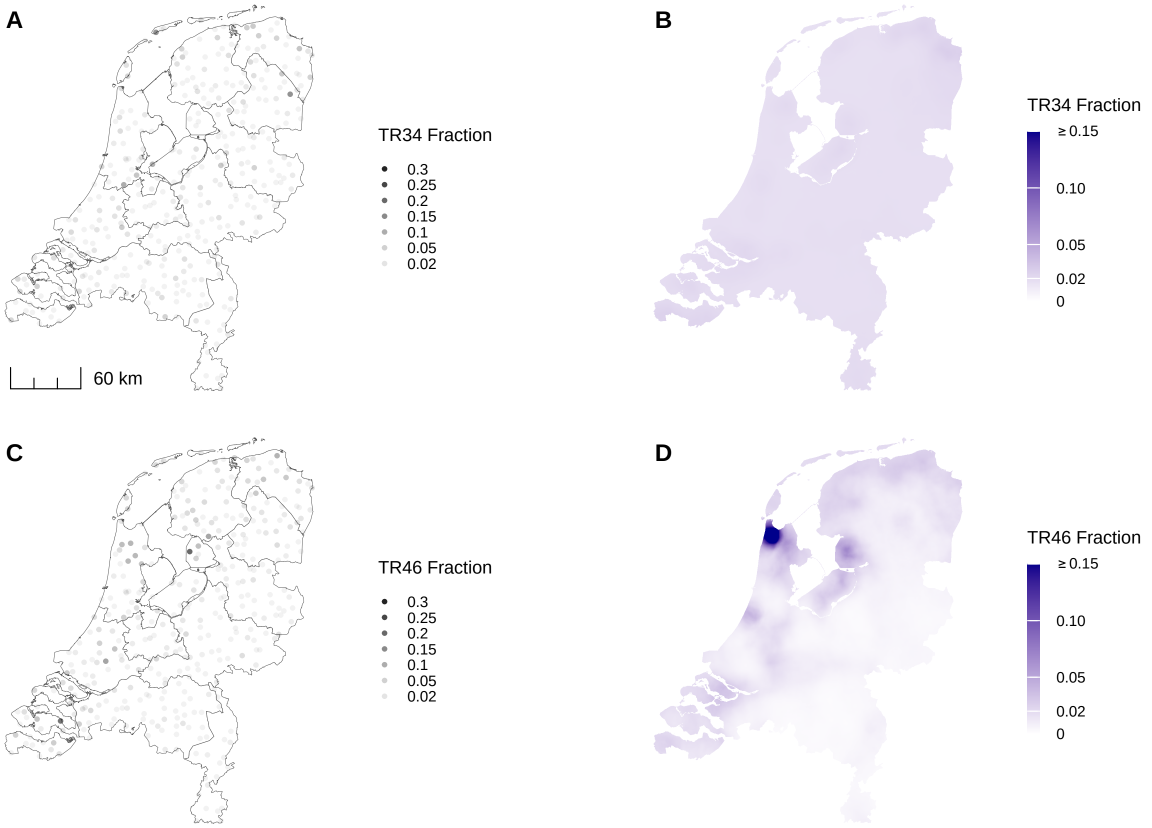

Fig. S5. Distribution of itraconazole-resistant TR_{34} and TR_{46} fractions. [A] The itraconazole-resistant TR_{34} fractions per sample (dots) as they were measured across the Netherlands. Each dot represents a sample. [B] Spatial prediction of the itraconazole-resistant TR_{34} fraction based on the corresponding land-use model (Table S15) [C] The itraconazole-resistant TR_{46} fractions per sample (dots) as they were measured across the Netherlands.[D] Spatial predictions of the itraconazole-resistant TR_{46} fraction based on the corresponding land-use model (Table S17).

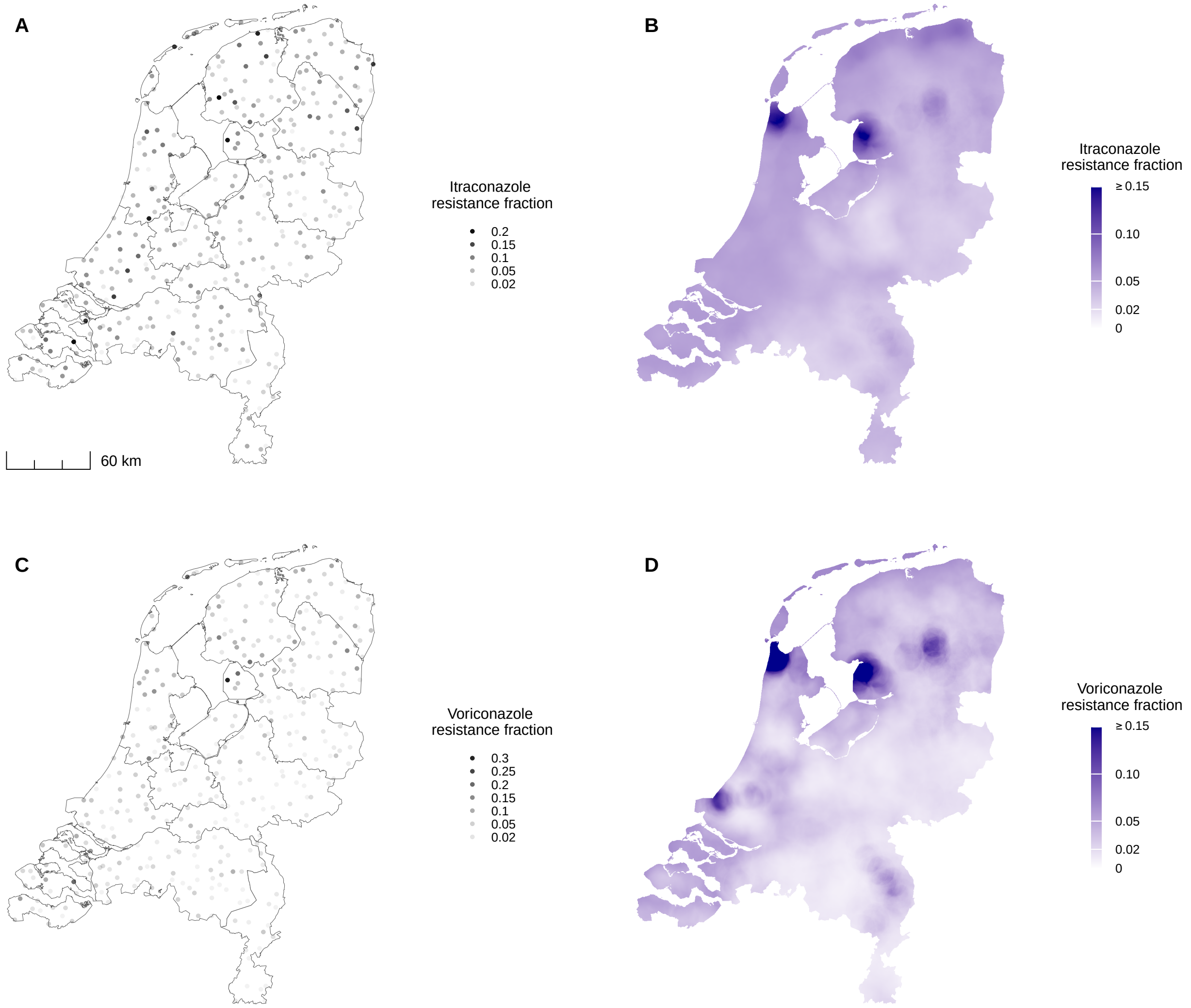

Fig. S6. Raw data [A, C] and spatial predictions [B, D] of the phenotypic itraconazole and voriconazole resistance fractions. [A] The itraconazole resistance fraction per sample as they were measured across the Netherlands. Each dot represents a sample. [B] Spatial prediction of the itraconazole resistance fraction based on the corresponding high-fungicide land-use model (Table S21) [C] The voriconazole resistance fractions per sample as they were measured across the Netherlands. Each dot represents a sample. [D] Spatial predictions of the voriconazole resistance fraction based on the corresponding high-fungicide land-use model (Table S23).

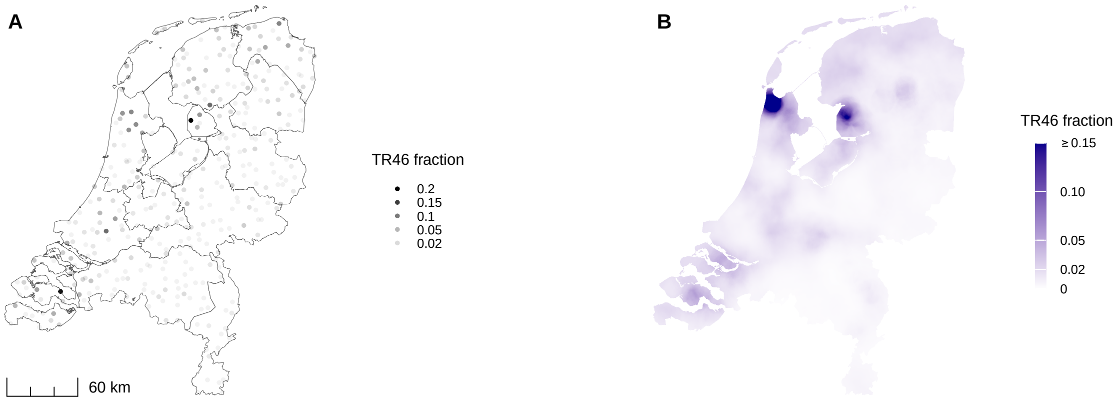

Fig. S7. Raw data and spatial predictions of the itraconazole-resistant TR_{46} fraction [A] The itraconazole-resistant TR_{46} fractions per sample as they were measured across the Netherlands. Each dot represents a sample. [B] Spatial predictions of the itraconazole-resistant TR_{46} fraction based on the minimal AIC high-fungicide land-use model of the data (Table S25).

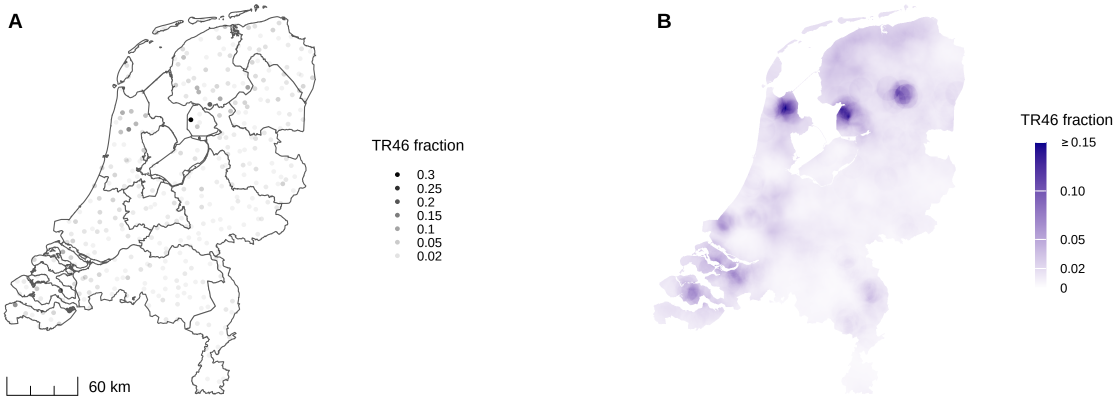

Fig. S8. Raw data and spatial predictions of the voriconazole-resistant TR_{46} fraction [A] The voriconazole-resistant TR_{46} fractions per sample as they were measured across the Netherlands. Each dot represents a sample. [B] Spatial predictions of the voriconazole-resistant TR_{46} fraction based on the minimal AIC high-fungicide land-use model of the data (Table S25).

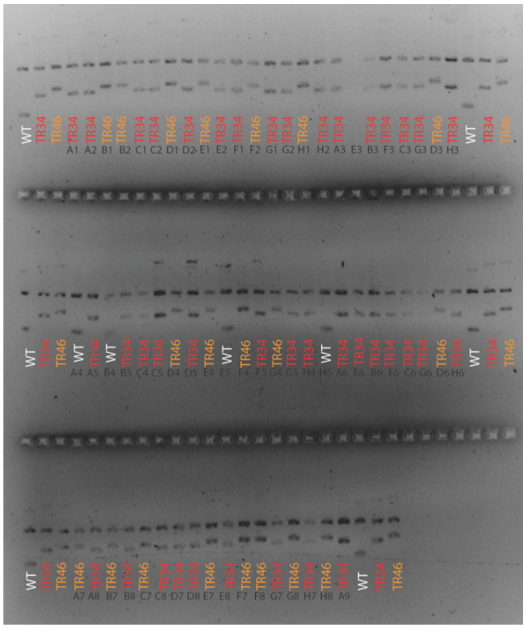

Fig. S9. Example of tandem repeat genotyping. A representative 0.5× TAE 3.5% (w/v) Low Melting PPC agarose gel (plate 3.6) after 100 min at 150 V, stained in 1 µg/mL EtBr in 0.5× TAE buffer for 1 hour. The first three slots contain the markers (1 µL) of WT, TR_{34}, and TR_{46}; in black, the location in the 96-well plate is indicated. Each slot contained 5 µL of the BglII digestion containing the PCR product.

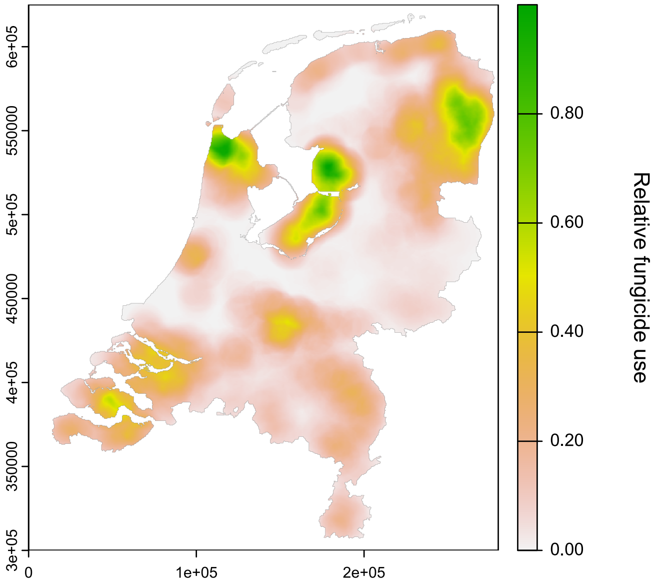

Fig. S10. Spatial distribution of estimated relative Dutch fungicide use. The distribution was made by weighing the spatial distributions (generated identically to those in Figure 2 and S4) of crops with available fungicide use data with their respective fungicide use in kg/hectare and summing all these distributions. The summed distribution was normalised to yield this relative fungicide use map. Note that this distribution assumes equal fungicide use across the entire area where a crop is grown.

Table S1. Overview of the 503 samples that were sent out for the citizen science project Schimmelradar (Fungal radar).

Status	Number	% of total
Sent out	503	100.0
No response	45	8.9
Failed[1]	46	9.1
Returned	412	81.9
Processed	365	72.6
Used in analysis	355	70.6

[1]Samples were scored as failed if the packages were reportedly sent back but never arrived, traps were reportedly deployed improperly (e.g. materials were damaged, exposure outside of sampling window, exposure <3 weeks, exposure >5 weeks)

Note to Supplemental Table S1:

After a Dutch media campaign involving television and radio, we received more than 8,600 registrations for the citizen science project Schimmelradar This high level of interest led us to scale up from an initially planned 300 to 503 selected participants. With 412 of the 503 packages successfully returned to the laboratory, we had a return rate of 81.9%. The most stated reasons why samples were not returned were logistical. Participants were not available during the sampling window, reportedly forgot or could not deploy the trap, lost the package or one of its critical components, or the package never arrived by post. Regarding the method, the most common issues were about how to install the sticky seals. A total of 15 participants either stuck the sticky side of the seal to the trap (upside down), stuck the cover of the seal rather than the seal itself to the trap for four weeks, or placed the cover of the seal back on the seal upside down. In the latter case, the cover cannot be removed from the seals and the sample cannot be processed anymore..

Table S2. Summary table of the *cyp51A* haplotypes of all itraconazole and voriconazole resistant genotyped *A. fumigatus* isolates.

	WT	TR_{34}	TR_{46}	$TR_{46^{*}3}$	$TR_{46^{*}4}$	Total
Itraconazole	152	590	365	4	0	1,111
Voriconazole	5	260	443	11	2	721
Total	157	850	808	15	2	1,832

Note to Table S2: We genotyped the *cyp51A* promoter variants of the colonies scored as resistant. Among all genotyped triazole-resistant CFUs, we observe an even ratio of the most common resistance haplotypes TR_{34} (n = 850) and TR_{46} (n = 808). Yet, the ratio of these haplotypes was significantly different ($\chi^2 = 16.7$, df = 1, $p = 4.378 \times 10^{-5}$) between those that were recovered from itraconazole or voriconazole plates; TR_{34} was dominant from itraconazole, while TR_{46} was dominant from voriconazole.

Table S3. All land-use classes grouped under the classes included during model selection.

Code	Land-use class (English translation)	Source	Included during model selection as
1	Agricultural grass	LGN	Grassland
2	Maize	LGN	Maize
3	Potatoes	LGN	Potatoes
5	Grains	LGN	Grains
8	Greenhouse horticulture	LGN	Greenhouses
9	Orchards	LGN	Fruits
10	Flower bulbs	LGN	Flower bulbs
11	Deciduous forest	LGN	Forest
12	Coniferous forest	LGN	Forest
16	Fresh water	LGN	Water
17	Salt water	LGN	Water
18	Buildings in primary built-up area	LGN	Urbanization
19	Buildings in secondary built-up area	LGN	Urbanization
20	Forest in primary built-up area	LGN	Forest
22	Forest in secondary built-up area	LGN	Forest
23	Grass in primary built-up area	LGN	Grassland
24	Bare ground in built-up area	LGN	Urbanization
26	Buildings in rural area	LGN	Urbanization
28	Grass in secondary built-up area	LGN	Grassland
40	Forest in raised bog area	LGN	Forest
43	Forest in marsh area	LGN	Forest
45	Naturally managed agricultural grasslands	LGN	Grassland
46	Grass in coastal area	LGN	Grassland
47	Other grass	LGN	Grassland
61	Tree nurseries	LGN	Tree farming
62	Fruit orchards	LGN	Fruits
251	Main infrastructure and rail track embankments	LGN	Urbanization
1098	Pears, planted before the current season	BRP	Apples/Pears
1096	Apples, planted before the current season	BRP	Apples/Pears
1095	Apples, planted during the current season	BRP	Apples/Pears
1097	Pears, planted during the current season	BRP	Apples/Pears
2014	Potatoes, consumption	BRP	Consumption potato
1003	Daffodil, bulbs and tubers	BRP	Daffodils/Hyacinths
982	Daffodil, other floriculture crops	BRP	Daffodils/Hyacinths
983	Daffodil, dried flowers	BRP	Daffodils/Hyacinths
998	Gladiolus, bulbs and tubers	BRP	Gladiolus
967	Gladiolus, other nursery crops	BRP	Gladiolus
968	Gladiolus, dried flowers	BRP	Gladiolus
999	Hyacinth, bulbs and tubers	BRP	Daffodils/Hyacinths
970	Hyacinth, other floriculture crops	BRP	Daffodils/Hyacinths
1018	Crested hyacinth, bulbs and tubers	BRP	Daffodils/Hyacinths
1002	Lily, bulbs and tubers	BRP	Lilies
979	Lily, other nursery crops	BRP	Lilies
980	Lily, dried flowers	BRP	Lilies
6664	Onions, red seed	BRP	Seed onions
2015	Potatoes, seed NAK	BRP	Seed potatoes
6660	Onions, yellow seed	BRP	Seed onions
2016	Potatoes, seed TBM	BRP	Seed potatoes
1933	Onions, seed and planting second year	BRP	Set onions
263	Onions, silver	BRP	Set onions
1934	Shallots	BRP	Set onions
2017	Potatoes, starch	BRP	Starch potatoes
1004	Tulip, bulbs and tubers	BRP	Tulips
985	Tulip, other nursery crops	BRP	Tulips

Note to Table S3: Only the more general land-use classes from the LGN (2022) dataset were used in the land-use models shown in Figure 1. The more specific high-fungicide crop classes from the BRP dataset were only included during model selection for the high-fungicide land-use models (see Table S20).

Table S4. Model summary of the averaged median CFU count weather + land-use model based on the 9 models with an AIC $\leq$ 2 greater than the lowest AIC model.

	Estimate (β)	Adjusted SE	z value	Pr($>|z|$)	Sum of Weights
Intercept	-128	23.9	5.35	1.00×10^{-7}	1
Distance to sea	1.84×10^{-6}	6.62×10^{-7}	2.78	0.00537	1
Forest	-1.22	0.431	2.82	0.00475	1
Grassland	-0.771	0.269	2.86	0.00423	1
Precipitation	-0.127	0.0359	3.53	0.000414	1
Relative humidity	0.151	0.0235	6.41	$< 2 \times 10^{-16}$	1
Temperature	0.425	0.0793	5.35	1.00×10^{-7}	1
Water	-1.23	0.239	5.15	3.00×10^{-7}	1
Grains	-0.93	0.612	1.52	0.128	0.559
Fruits	1.84	1.62	1.13	0.258	0.236
Flower bulbs	-1.28	1.65	0.775	0.438	0.172
Urbanization	0.197	0.291	0.677	0.498	0.157
Greenhouses	-1.12	2.37	0.473	0.636	0.0721

Note to Table S4: The estimate (β) represents the log-expected counts and indicates the size and direction of the slope of the correlation between the explanatory and response variables in a negative binomial model. The "Sum of Weights" indicates how frequently each variable occurred among the 9 best-fitting models. A higher value reflects greater consistency and robustness in predictive effect across models.

Table S5. Summary of best-fitting (lowest AIC) model of median CFU counts (weather + land-use), used in prediction plot (Figure 1G). Columns as in Table S4.

	Estimate (β)	Std. Error	z value	Pr($>|z|$)
Intercept	-126	22.8	-5.53	3.21×10^{-8}
Distance to sea	1.76×10^{-6}	6.49×10^{-7}	2.71	0.00665
Forest	-1.38	0.405	-3.41	0.000639
Grains	-0.897	0.603	-1.49	0.137
Grassland	-0.875	0.251	-3.48	0.000502
Precipitation	-0.134	0.034	-3.96	7.65×10^{-5}
Relative humidity	0.151	0.0231	6.55	5.70×10^{-11}
Temperature	0.418	0.0753	5.55	2.87×10^{-8}
Water	-1.29	0.217	-5.96	2.58×10^{-9}

Table S6. Summary of the averaged itraconazole resistance land-use model of the 33 models with AIC $\leq$ 2 greater than the minimal AIC model.

	Estimate (β)	Adjusted SE	z value	Pr($>|z|$)	Sum of Weights
Intercept	-2.8780505	0.3387458	8.50	< 0.0001	1
Maize	-2.96	1.38	2.14	0.032	0.934
Forest	-1.79	0.715	2.51	0.0122	0.931
Flower bulbs	5.56	2.94	1.89	0.0588	0.862
Onions	6.72	3.92	1.72	0.086	0.675
Tree Farming	-5.89	4.64	1.27	0.204	0.359
Potatoes	1.65	1.24	1.33	0.185	0.279
Urbanization	-0.755	0.65	1.16	0.245	0.231
Water	0.74	0.673	1.10	0.272	0.166
Grassland	0.547	0.816	0.67	0.503	0.135
Greenhouses	5.17	5.31	0.974	0.330	0.135
Fruit	-2.8	3.8	0.737	0.461	0.0497
Grains	0.656	1.35	0.484	0.628	0.0414

Note to Table S6: The estimate (β) represents the log-odds of the effect size and indicates the size and direction of the slope of the correlation between the explanatory and response variables in a betabinomial model. The "Sum of Weights" indicates how frequently each variable occurred among the 9 best-fitting models. A higher value reflects greater consistency and robustness in predictive effect across models.

Table S7. Summary of lowest AIC itraconazole resistance land-use model used in prediction plot (Figure 1H). Columns as in Table S6.

	Estimate (β)	Std. Error	z value	Pr($>|z|$)
Intercept	−2.83	0.0884	−32.1	7.90×10^{-226}
Flower bulbs	5.18	2.94	1.76	0.0786
Forest	−1.88	0.704	−2.67	0.00762
Maize	−2.94	1.3	−2.26	0.0236
Onions	6.54	3.49	1.88	0.0607

Table S8. Model summary of the averaged voriconazole resistance land-use model of the 11 models with AIC $\leq$ 2 greater than the lowest AIC model. Columns as in Table S6.

	Estimate (β)	Adjusted SE	z value	Pr($>|z|$)	Sum of Weights
Intercept	−3.77	0.952	3.97	7.30×10^{-5}	
Flower bulbs	11.5	3.32	3.47	0.000522	1
Urbanization	−3.76	1.2	3.13	0.00173	1
Greenhouses	20.2	6.38	3.16	0.00158	1
Potatoes	5.74	1.82	3.16	0.00159	1
Maize	−4.37	2.24	1.95	0.0509	0.819
Grassland	1.76	0.751	2.34	0.0193	0.759
Water	1.8	0.693	2.6	0.00943	0.692
Grains	−3.06	1.83	1.67	0.0942	0.387
Forest	−1.77	1.48	1.2	0.232	0.373
Fruit	−4.58	5.69	0.804	0.422	0.162
Onions	3.56	5.18	0.688	0.491	0.131
Tree Farming	0.484	5.8	0.0835	0.933	0.0598

Table S9. Summary of lowest AIC voriconazole resistance phenotype land-use model used in prediction plot (Figure 1I). Columns as in Table S6.

	Estimate (β)	Std. Error	z value	Pr($>|z|$)
Intercept	−4.16	0.492	−8.47	2.55×10^{-17}
Flower bulbs	12.3	2.89	4.27	1.98×10^{-5}
Urbanization	−3.66	1.13	−3.24	0.0012
Grassland	1.81	0.543	3.33	0.000861
Greenhouses	21.3	6.05	3.52	0.000433
Maize	−3.58	2.11	−1.7	0.089
Potatoes	6.06	1.52	4	6.33×10^{-5}
Water	1.61	0.6	2.68	0.00739

Table S10. Model summary of the averaged voriconazole-resistant TR46 land-use model of the 14 models with AIC $\leq$ 2 greater than the lowest AIC model. Columns as in Table S6.

	Estimate (β)	Adjusted SE	z value	Pr($>|z|$)	Sum of Weights
Intercept	-1.34	1.66	0.808	0.419	1
Urbanisation	-6.05	1.55	3.91	9.15×10^{-5}	1
Maize	-9.72	3.40	2.86	0.00421	1
Grains	-7.56	3.99	1.90	0.0579	0.925
Flower bulbs	10.4	4.79	2.16	0.0304	0.877
Forest	-5.90	2.49	2.37	0.0177	0.871
Water	-3.07	1.47	2.09	0.0369	0.871
Grassland	-1.74	1.82	0.959	0.337	0.815
Greenhouses	17.6	9.28	1.89	0.0584	0.722
Potatoes	2.66	2.37	1.12	0.263	0.263
Tree Farming	-6.93	8.40	0.825	0.41	0.122
Onions	-5.33	7.71	0.691	0.489	0.106
Fruit	-3.03	6.75	0.449	0.653	0.0497

Table S11. Summary of lowest AIC voriconazole-resistant TR46 land-use model used in prediction plot (see Figure 1K). Columns as in Table S6.

	Estimate (β)	Std. Error	z value	Pr(> $\|z\|$)
Intercept	-0.949	1.04	-0.914	0.361
Flower bulbs	8.98	4.39	2.05	0.0408
Urbanization	-6.51	1.42	-4.58	4.58×10^{-6}
Forest	-5.85	2.04	-2.86	0.0042
Grains	-7.70	3.29	-2.34	0.0194
Grassland	-1.82	1.11	-1.64	0.101
Greenhouses	15.3	8.99	1.71	0.0879
Maize	-9.94	3.06	-3.25	0.00117
Water	-3.10	1.13	-2.73	0.00633

Table S12. Model summary of the averaged voriconazole-resistant TR34 land-use model of the 75 models with AIC $\leq$ 2 greater than the lowest AIC model. Columns as in Table S6.

	Estimate (β)	Adjusted SE	z value	Pr(> $\|z\|$)	Sum of Weights
Intercept	-3.92	0.646	6.07	0	1
Onions	-25.4	13	1.96	0.0504	0.924
Fruit	-18.5	10.8	1.72	0.0859	0.861
Urbanization	-3.73	1.66	2.24	0.025	0.795
Maize	-6.9	3.11	2.21	0.0268	0.744
Tree Farming	11.6	7.34	1.58	0.115	0.437
Grains	3.29	2.08	1.58	0.113	0.415
Water	1.41	0.644	2.18	0.0291	0.349
Greenhouses	14.4	10.5	1.37	0.17	0.341
Grassland	-0.87	0.7	1.24	0.214	0.22
Flower bulbs	-9.17	9.31	0.985	0.325	0.205
Potatoes	2.11	2.5	0.845	0.398	0.102
Forest	-0.717	1.53	0.47	0.639	0.0435

Table S13. Summary of lowest AIC voriconazole-resistant TR34 land-use model used in prediction plot (Figure 1J). Columns as in Table S6.

	Estimate (β)	Std. Error	z value	Pr(> $\|z\|$)
Intercept	-3.59	0.249	-14.4	4.01×10^{-47}
Urbanization	-3.9	1.36	-2.87	0.00408
Fruit	-18.5	10.5	-1.76	0.0791
Maize	-8.38	2.6	-3.22	0.00129
Onions	-21	10.5	-2	0.0454
Tree farming	12.2	7.08	1.72	0.0857

Table S14. Model summary of the averaged itraconazole-resistant TR34 land-use model of the 28 models with AIC $\leq$ 2 greater than the lowest AIC model. Columns as in Table S4.

	Estimate (β)	Adjusted SE	z value	Pr(> $\|z\|$)	Sum of Weights
Intercept	-3.98	0.139	28.7	0	1
Forest	1.52	1.21	1.26	0.209	0.412
Grains	-0.982	0.862	1.14	0.255	0.258
Grassland	0.316	0.471	0.672	0.502	0.0785
Flower bulbs	0.524	1.36	0.386	0.699	0.0955
Water	-4.08	5.68	0.717	0.473	0.11
Potatoes	4.28	7.04	0.607	0.544	0.0989
Greenhouses	-0.665	1.57	0.424	0.671	0.0654
Maize	1.47	5.71	0.257	0.797	0.0643
Onions	-0.194	0.42	0.462	0.644	0.0967
Fruit	-2.43	4.96	0.489	0.625	0.0938
Tree Farming	-0.352	5.2	0.0677	0.946	0.0591
Urbanisation	0.195	0.833	0.234	0.815	0.0608

Table S15. Summary of lowest AIC itraconazole-resistant TR34 land-use model used in prediction plot (Table S2B). Columns as in Table S4.

	Estimate (β)	Std. Error	z value	Pr($>|z|$)
Intercept	-4.03	0.0769	-52.4	0
Grains	1.45	1.11	1.31	0.192

Table S16. Model summary of the averaged itraconazole-resistant TR46 land-use model of the 13 models with AIC $\leq$ 2 greater than the lowest AIC model. Columns as in Table S4.

	Estimate (β)	Adjusted SE	z value	Pr($>|z|$)	Sum of Weights
Intercept	-3.21	0.486	6.6	0	1
Flower bulbs	14.5	3.63	3.98	6.81×10^{-5}	1
Urbanization	-4.24	1.39	3.04	0.00235	1
Forest	-4.07	1.52	2.68	0.00737	1
Maize	-10.2	3.41	3	0.00269	1
Water	-1.01	0.613	1.66	0.0976	0.622
Grassland	0.768	0.639	1.2	0.23	0.284
Onions	7.84	6.45	1.22	0.224	0.28
Greenhouses	9.72	9.59	1.01	0.311	0.142
Fruit	5.79	7.05	0.821	0.412	0.0744
Tree Farming	-1.02	1.56	0.651	0.515	0.0672
Grains	-0.68	1.68	0.406	0.685	0.0588
Potatoes	-3.87	9.84	0.393	0.694	0.0588

Table S17. Summary of lowest AIC itraconazole resistant TR46 land-use model used in prediction plot (Table S2D). Columns as in Table S4.

	Estimate (β)	Std. Error	z value	Pr($>|z|$)
Intercept	-2.95	0.321	-9.17	4.74×10^{-20}
Flower bulbs	14.3	3.54	4.05	5.21×10^{-5}
Urbanization	-4.43	1.27	-3.49	0.000486
Forest	-4.37	1.43	-3.05	0.00228
Maize	-11.1	3.24	-3.42	0.000617
Water	-1	0.58	-1.72	0.0846

Table S18. Copy of the most recent (2020) available fungicide use data per crop class of the Dutch statistics bureau (CBS). Data source: ***https://opendata.cbs.nl/#/CBS/nl/dataset/85130NED/table?ts=1729866150339***. Data are sorted by dosage per year per hectare of fungicide use.

Crop (English translation)	Surface with crop protection (ha)	Percentage with crop protection (%)	Total use (kg)	Use/ha/year (kg/ha)	Dosage/ha/year (kg/ha)	Included in high-fungicide model
Roses, greenhouse	198	97.1	7034	34.5	35.5	N
Lilies (bulb)	5021	95.0	140761	26.6	28.0	Y
Apples	5889	95.7	116161	18.9	19.7	Y
Tulips, open field	14897	100.0	291128	19.5	19.5	Y
Pears	9825	98.2	177628	17.8	18.1	Y
Gladioli	803	98.9	11239	13.8	14.0	Y
Daffodils	1434	94.2	18511	12.2	12.9	Y
Hyacinths	1408	98.8	16649	11.7	11.8	Y
Fruit trees	1413	89.2	13861	8.8	9.8	Y
Starch potatoes	45104	100.0	427675	9.5	9.5	Y
Seed and planting onions	9043	96.2	78796	8.4	8.7	Y
Seed onions	26559	97.4	209858	7.7	7.9	Y
Cucumbers, greenhouse	559	93.9	4369	7.3	7.8	N
Chrysanthemums, greenhouse	396	89.0	3045	6.8	7.7	N
Gerbera, greenhouse	145	88.5	1091	6.7	7.5	N
Strawberries, greenhouse	490	97.2	2998	5.9	6.1	N
Potatoes for consumption, total	63126	82.3	320061	4.2	5.1	Y
Seed potatoes, total	42697	97.5	214655	4.9	5.0	Y
Potted plants, greenhouse	663	69.2	3295	3.4	5.0	N
Total crops/cultivation sectors	449326	63.1	2193633	3.1	4.9	N
Strawberries, production	976	96.8	4560	4.5	4.7	N
Perennials	1111	74.1	4008	2.7	3.6	N
Leafy plants, greenhouse	327	71.0	1057	2.3	3.2	N
Tomatoes, greenhouse	1522	81.3	4912	2.6	3.2	N
Leeks	2110	93.0	5000	2.2	2.4	N
Floriculture crops, total	2548	72.0	5125	1.4	2.0	N
Bell pepper, greenhouse	921	60.1	1742	1.1	1.9	N
Cabbage, total	2015	76.0	3499	1.3	1.7	N
Brussels sprouts	2715	94.1	4209	1.5	1.6	N
Asparagus, total	2002	58.6	3241	0.9	1.6	N
Winter carrots	4724	72.0	4246	0.6	0.9	N
Forest and hedge planting	2007	73.8	1403	0.5	0.7	N
Sugar beets	66728	81.9	42085	0.5	0.6	N
Dwarf green beans	1916	66.3	1207	0.4	0.6	N
Forest and washing carrots	2126	70.4	1102	0.4	0.5	N
Wheat, winter	85842	92.5	34688	0.4	0.4	N
Avenue and park trees	3681	77.4	1367	0.3	0.4	N
Chicory root	1279	40.8	469	0.1	0.4	N
Barley, summer	21052	72.7	6690	0.2	0.3	N
Wheat, summer	11791	70.3	3777	0.2	0.3	N
Chicory	1681	43.6	309	0.1	0.2	N
Rapeseed, total	581	34.5	124	0.1	0.2	N
Maize, silage maize	0	0.0	0	0.0	0.0	N

Note to Table S18: Only crops with a dosage per year per hectare of $\geq$ 5 kg/ha were included in the high-fungicide land-use models. Greenhouse crops were excluded due to their small and spatially clustered distributions.

Table S19. Model summary of the averaged phenotypic itraconazole resistance high-fungicide land-use model of the 37 models with AIC ≤ 2 greater than the minimal AIC model. Columns as in Table S6.

	Estimate (β)	Adjusted SE	z value	Pr($>|z|$)	Sum of Weights
Intercept	-2.76	0.106	26.00	$< 2 \times 10^{-16}$	1
Forest	-1.92	0.744	2.58	0.00981	1
Maize	-4.09	1.37	2.99	0.00281	1
Lilies	39.7	16.4	2.42	0.0156	0.970
Seed potatoes	3.42	2.35	1.45	0.146	0.475
Set Onions	16.3	12.8	1.27	0.204	0.244
Seed Onions	5.02	4.40	1.14	0.254	0.177
Tree farming	-4.78	4.56	1.05	0.295	0.167
Starch potatoes	0.914	1.15	0.80	0.427	0.110
Apples/Pears	4.03	4.59	0.88	0.380	0.0933
Urbanisation	-0.446	0.622	0.72	0.473	0.0893
Tulips	4.03	5.31	0.76	0.449	0.0705
Gladiolus	166	136	1.23	0.220	0.0554
Grassland	-0.25	0.291	0.86	0.391	0.0535
Grains	0.436	0.886	0.49	0.622	0.043
Water	0.199	0.321	0.62	0.535	0.0233
Greenhouses	2.42	4.70	0.52	0.607	0.0219
Daffodils/Hyacinths	-0.29	16.6	0.02	0.986	0.0193

Table S20. Summary of lowest AIC phenotypic itraconazole resistance high-fungicide land-use model used in prediction plot (Table S10B). Columns as in Table S6.

	Estimate (β)	Std. Error	z value	Pr($>|z|$)
Intercept	-2.78	0.0869	-32.00	1.19×10^{-224}
Forest	-1.84	0.731	-2.52	0.0117
Lilies	36.8	15.8	2.33	0.0196
Maize	-4.07	1.33	-3.07	0.00212
Seed potatoes	3.44	2.23	1.54	0.123

Table S21. Model summary of the averaged phenotypic voriconazole resistance high-fungicide land-use model of the 62 models with AIC ≤ 2 greater than the minimal AIC model. Columns as in Table S6.

	Estimate (β)	Adjusted SE	z value	Pr($>|z|$)	Sum of Weights
Intercept	-2.71	0.508	5.33	1.00×10^{-7}	1
Urbanization	-3.72	1.01	3.67	0.000244	1
Greenhouses	16.9	6.02	2.81	0.00491	1
Lilies	79.4	22.4	3.55	0.000391	1
Maize	-6.46	1.94	3.32	0.000887	1
Forest	-2.72	0.99	2.75	0.00595	0.911
Starch potatoes	3.28	1.95	1.69	0.0918	0.578
Seed potatoes	5.55	3.4	1.64	0.102	0.525
Daffodils/Hyacinths	-28.9	23.6	1.23	0.22	0.487
Grains	-2.04	1.48	1.37	0.169	0.4
Set Onions	28.6	21.1	1.35	0.176	0.26
Seed Onions	-5.74	6.86	0.837	0.403	0.153
Gladiolus	179	183	0.975	0.33	0.129
Grassland	1.31	0.813	1.62	0.106	0.116
Water	1.22	0.948	1.28	0.199	0.116
Tulips	5.06	6.08	0.832	0.405	0.0914
Apples/Pears	-4.25	6.97	0.611	0.541	0.0529
Tree farming	-1.72	5.79	0.297	0.767	0.0347

Table S22. Summary of lowest AIC phenotypic voriconazole resistance high-fungicide land-use model used in prediction plot (Table S10D). Columns as in Table S6.

	Estimate (β)	Std. Error	z value	Pr($>|z|$)
Intercept	-2.55	0.12	-21.1	3.81×10^{-99}
Urbanization	-3.96	0.89	-4.44	8.79×10^{-6}
Forest	-2.72	0.948	-2.87	0.00408
Greenhouses	16.8	5.94	2.83	0.00469
Lilies	90.7	16.1	5.62	1.88×10^{-8}
Maize	-6.87	1.7	-4.04	5.43×10^{-5}

Table S23. Model summary of the averaged itraconazole resistant TR_{46} high-fungicide land-use model of the 70 models with AIC $\leq$ 2 greater than the lowest AIC model. Columns as in Table S6.

	Estimate (β)	Adjusted SE	z value	Pr($>|z|$)	Sum of Weights
Intercept	-3.8	0.611	6.21	0	1
Lilies	77.7	31.5	2.47	0.0135	1
Maize	-11.3	3.48	3.26	0.00113	1
Urbanisation	-3.08	1.38	2.24	0.0253	0.973
Forest	-3.57	1.64	2.18	0.0294	0.964
Apple/Pear	15.3	8.26	1.85	0.064	0.8
Tulips	13.7	8.08	1.7	0.09	0.651
Grassland	1.11	0.788	1.4	0.161	0.575
Seed potatoes	6.53	4.12	1.58	0.113	0.537
Set Onions	40.7	26.4	1.54	0.123	0.443
Water	-0.103	1.34	0.0769	0.939	0.186
Daffodils/Hyacinths	24.9	28.6	0.869	0.385	0.125
Seed Onions	-8.33	9.1	0.915	0.36	0.0999
Greenhouses	7.68	9.68	0.793	0.428	0.0993
Grains	1.09	2.75	0.398	0.691	0.0633
Gladiolus	-182	282	0.646	0.518	0.058
Tree farming	-5.91	10.6	0.557	0.578	0.0468

Table S24. Summary of lowest AIC itraconazole resistant TR_{46} high-fungicide land-use model used in prediction plot (Table S13B). Columns as in Table S6.

	Estimate (β)	Std. Error	z value	Pr($>|z|$)
Intercept	-3.93	0.288	-13.6	3.09×10^{-42}
Apples/Pears	13.5	8.39	1.61	0.108
Urbanization	-3.53	1.27	-2.78	0.0055
Forest	-3.18	1.53	-2.07	0.0381
Grass	1.13	0.591	1.92	0.0552
Lilies	78.3	29.6	2.64	0.00828
Maize	-12.1	3.26	-3.71	0.000204
Set Onions	37	24.2	1.53	0.126
Tulips	14.1	7.63	1.85	0.065

Table S25. Model summary of the averaged voriconazole resistant TR_{46} high-fungicide land-use model of the 109 models with AIC ≤ 2 greater than the lowest AIC model. Columns as in Table S6.

	Estimate (β)	Adjusted SE	z value	Pr($> \|z\|$)	Sum of Weights
Intercept	-2.79	1.12	2.49	0.0127	1
Urbanisation	-4.93	1.58	3.11	0.00185	1
Maize	-9.92	3.08	3.22	0.00129	1
Set Onions	65.0	22.9	2.84	0.00457	1
Grains	-5.65	2.75	2.05	0.0402	0.857
Forest	-3.73	1.80	2.08	0.0378	0.839
Greenhouses	17.2	8.82	1.95	0.0515	0.837
Water	-1.68	1.12	1.50	0.133	0.791
Gladiolus	432	230	1.88	0.0603	0.731
Lilies	74.6	34.4	2.17	0.0299	0.698
Seed Onions	-15.5	10.8	1.44	0.15	0.409
Grassland	0.534	1.90	0.281	0.778	0.351
Seed potatoes	9.26	5.23	1.77	0.0769	0.339
Daffodils/Hyacinths	-49.7	36.2	1.37	0.17	0.284
Starch potatoes	3.36	2.58	1.30	0.193	0.224
Tulips	10.3	8.06	1.27	0.203	0.122
Tree farming	-4.37	8.46	0.517	0.605	0.0138
Apples/Pears	-4.06	8.92	0.455	0.649	0.0133

Table S26. Summary of lowest AIC voriconazole resistant TR_{46} high-fungicide land-use model used in prediction plot (Table S15B). Columns as in Table S6.

	Estimate (β)	Std. Error	z value	Pr($> \|z\|$)
Intercept	-2.65	0.408	-6.49	8.53×10^{-11}
Urbanization	-5.02	1.49	-3.37	0.000765
Daffodil/Hyacinth	-53.4	35.2	-1.52	0.13
Forest	-3.30	1.33	-2.48	0.0131
Gladiolus	361	225	1.61	0.108
Grains	-4.92	1.96	-2.51	0.0121
Greenhouses	16.8	8.54	1.97	0.0494
Lilies	57.9	34.3	1.69	0.0911
Maize	-10.0	2.87	-3.49	0.000475
Seed Onions	-19.5	10.1	-1.93	0.0539
Seed potatoes	8.99	5.00	1.80	0.0721
Set Onions	69.8	20.1	3.47	0.000521
Water	-1.55	0.623	-2.49	0.0129

Table S27. Overview of model fit (AIC) and predictive accuracy (RSME). We compare the null models for each response variable to the minimal AIC or best model identified during model selection across our designated parameter space. The null model or intercept model assumes the response equals the overall mean (proportion or count). We consider ΔAIC < -10 and ΔRSME $> 5\%$ as meaningful improvements.

Response variable	Model selection parameters	AIC null	AIC best	ΔAIC	RSME null	RSME best	ΔRSME (%)
Itraconazole resistance	Land use (LGN)	1658.5	1624.4	−34.1	0.0377	0.0358	5.04
Voriconazole resistance	Land use (LGN)	1505.0	1410.5	−94.5	0.0389	0.0351	9.77
Itraconazole resistant TR_{34}	Land use (LGN)	1184.9	1185.3	0.4	0.0204	0.0204	0.0835
Itraconazole resistant TR_{46}	Land use (LGN)	975.2	910.0	−65.2	0.0231	0.0208	10.0
Voriconazole resistant TR_{34}	Land use (LGN)	805.3	790.7	−14.6	0.0182	0.0176	3.34
Voriconazole resistant TR_{46}	Land use (LGN)	1077.5	1032.9	−44.6	0.0278	0.0259	6.50
Itraconazole resistant WT	Land use (LGN)	574.9	571.9	−3.0	0.0132	0.0132	0.16
Itraconazole resistance	LGN + high-fungicide land use (BRP)	1658.5	1621.2	−37.3	0.0377	0.0353	6.37
Voriconazole resistance	LGN + high-fungicide land use (BRP)	1505.0	1405.5	−99.5	0.0389	0.0335	13.9
Itraconazole resistant TR_{46}	LGN + high-fungicide land use (BRP)	975.2	906.4	−68.85	0.0231	0.0211	8.73
Voriconazole resistant TR_{46}	LGN + high-fungicide land use (BRP)	1077.5	1024.8	−52.7	0.0278	0.0257	7.52
Itraconazole resistance	Fungicide use	1658.5	1644.6	−13.9	0.0377	0.0372	1.20
Voriconazole resistance	Fungicide use	1505.0	1475.1	−29.9	0.0389	0.0371	4.58
Median CFU counts per trap	LGN + weather data (AgERA5)	3333.7	3252.3	−81.4	28.2	25.9	8.16
