# Supplementary material for: Land use drives drug resistance in an airborne human fungal pathogen
Source: ISME J. 2025 Nov 6;19(1):wraf246. doi: 10.1093/ismejo/wraf246 (PMC12646274; doi:10.1093/ismejo/wraf246)
Supplement: ISME_s_text_Schimmelradar_(30-10-25)_wraf246 [file isme_s_text_schimmelradar_(30-10-25)_wraf246.docx]

Supplementary Text for:

**Land use drives drug resistance in an airborne human**

**fungal pathogen**

**This file includes:**

Supplementary Text A and B

Supplementary Text

**Supplementary Text A: Press release citizen science project**

**(English version, the original Dutch text is available on request)**

**
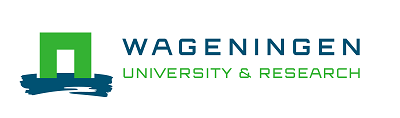
**

Article of Wageningen University & Research 6^th^ of September 2023

**Call: Help scientists from Wageningen with collecting fungal spores.**

**Scientists from Wageningen are calling upon people from all over The Netherlands for assistance in October and November to help them map the presence of spores of the fungus *Aspergillus fumigatus* in the air. The results will help the researchers understand how the fungus develops resistance to medical antifungals and how this resistance is spreading across The Netherlands. More information about this is essential, as doctors are facing increasing difficulty in treating vulnerable patients by treating fungal infections. Assisting with collecting the fungal spores can easily be done and is not time-consuming for the participants.**

Fungi are everywhere around us, and many of these fungi spread via spores in the air. So does *Aspergillus fumigatus,* a fungus that lives in compost but can also invade the lungs of already seriously ill patients. Infection with this fungus can lead to a deadly disease called aspergillosis. The most important drugs used to treat this infection contain azoles, but the increase in azole resistance in this fungus results in limited effectiveness of the drug.

**Azoles**Azoles are used in multiple industries, such as the clinical treatment against fungal infections, but also to protect crops against plant pathogenic fungi or in paint to conserve wood. There is a growing body of evidence that the use of azoles in the environment leads to a decrease in the effectiveness of the drugs that contain azoles. Azole resistance in the fungus *Aspergillus fumigatus* leads to infections with this fungus becoming increasingly difficult to treat. This drastically increases mortality in patients.

**Hazard for vulnerable patients**Nearly everyone breathes the spores of *Aspergillus fumigatus* daily, yet most people do not get sick. People with a weakened or suppressed immune system, resulting from organ transplants or chemotherapy, or with impaired lung function because of Cystic Fibrosis or smoker’s lung, are a lot more susceptible to this infection. To be able to keep on treating these patients, it is essential to limit the transmission of azole-resistant *Aspergillus fumigatus*. To determine where a lot of azole-resistant *Aspergillus fumigatus* originates, it is important to figure out how much azole-resistant *Aspergillus fumigatus* is present in the air.

**How can you participate in this study?**In the fall, scientists or WUR want to map the percentage of azole resistance in *Aspergillus fumigatus* in the air in The Netherlands. Here, the focus will be on the baseline of the resistance in the air and if there are regional differences to enable further research about specific sources of increased resistance.


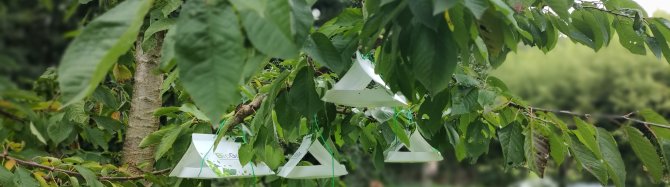


The WUR scientists, who collaborate with the RIVM and Radboud UMC, need assistance to measure all these fractions. Therefore, they are looking for participants who are willing to put up a trap (see picture) for 4 weeks near their house. This trap – together with the simple instructions, will be sent by mail, and can be readily returned by envelope.

**Supplementary text B: Participant instructions**

**(English version, the original Dutch text is available on request)**

**Air sampling – Wageningen University & Research – October to November 2023**

Dear Participant,

First of all, thank you for participating in Schimmelradar! A large-scale measurement like this would not be possible without the help of many volunteers like you. In this project, we will be measuring the baseline levels of azole resistance in *Aspergillus fumigatus* in the Netherlands. By collecting these data, we can begin to identify if and where in the Netherlands there is increased azole resistance. If you would like to learn more about the importance of preventing azole resistance in *Aspergillus fumigatus*, you can read more at Schimmelradar.nl or email us at schimmel.radar@wur.nl. For updates, you can follow us on X (formerly Twitter): @Schimmelradar.

**Contents of this envelope**
In this envelope, you will find everything you need to perform the air measurements. In addition to this letter and the information sheet, the envelope contains the following items:

• A folded delta trap secured with rubber bands.

• A piece of string\rope.

• A resealable zip-lock bag containing:

- 3 sticky seals from the brand BioRad
- 6-7 white adhesive putties
- A data sheet

• An instruction sheet explaining how to use the items listed above in the measurements.

If your package is incomplete, please email [schimmel.radar@wur.nl](mailto:schimmel.radar@wur.nl).

**When does the project take place?**

The traps will be hung outside throughout the Netherlands between October 1 and November 12. The traps can be hung between October 1 and October 15. It is important that the trap remains outside for exactly 4 weeks. When you take the trap down will depend on when you initially set it up.

**How do I perform the air sampling?**
How to do the air sampling is explained in the videos (see the QR code on the instruction sheet) and on the instruction sheet itself. After four weeks, the sticky seals can be returned. After taking the sample and before posting it, it is important to store the sticky seals in a dark, dry place at room temperature.

For our research, it is crucial that the following items are returned:

- The data sheet
- The three BioRad sticky seals

Pack everything in the plastic zip-lock bag before placing it in the return envelope.

**Return your sample to:**

Schimmelradar
Laboratorium voor Erfelijkheidsleer (Bodenr. 44)
Antwoordnummer 118
6700 VB Wageningen

Please return the materials **by December 1, 2023**. The first results will be announced in early 2024.

**Visibility of the research**
In addition to contributing to the sampling effort, you can also help increase the visibility of this research. Within the project, we are mainly active on Twitter. You can find us there under @Schimmelradar, and we use the hashtag #SporenOpsporen.

The explanation on this sheet can also be found in video format under these QR-codes:

**
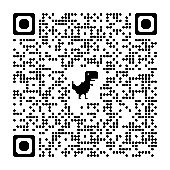

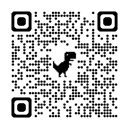
Deploying the trap:** **Returning the sticky seals:**

**Deploying the delta trap**

1. Check the contents of your air sampling package


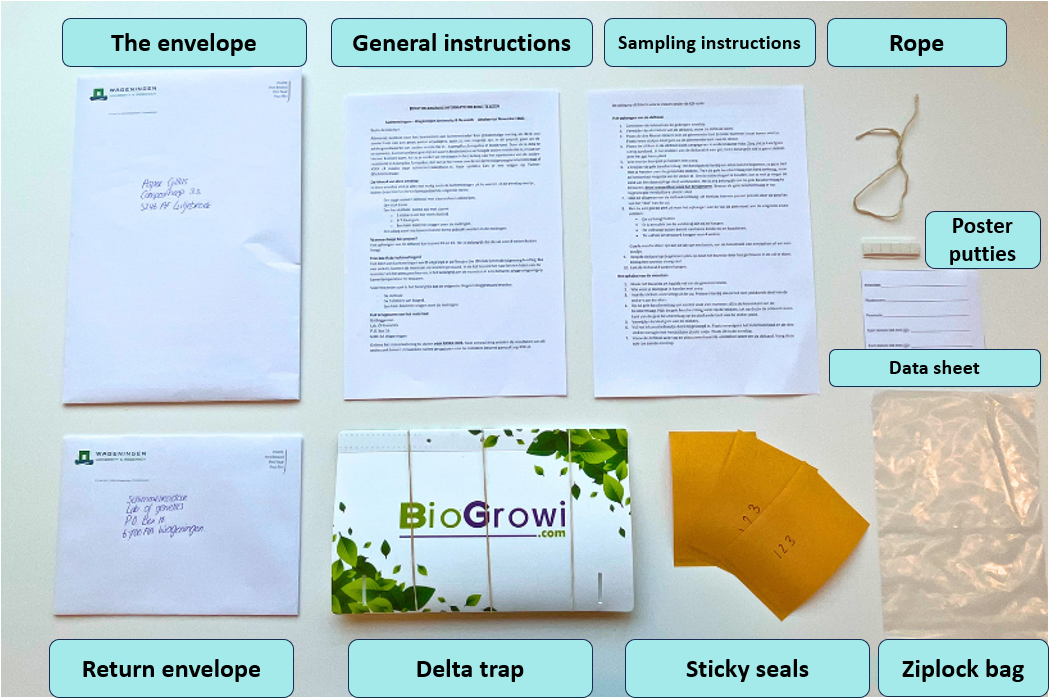


1. Remove the rubber bands from the delta trap, and unfold it.
2. Place the three Bio-Rad sticky seals with “shiny”, unenumerated side facing up. Place two poster putties on this side of the seal.
3. Fold on side of the floor of the delta trap up; make sure to anchor the corners of the floor in the slits in roof of the trap.
4. Place the stickers in the delta trap as shown in the picture below, making sure the furthest sticker is placed horizontally. Firmly press the seals onto the trap such that the adhesive putties stick well. There is a hole in the middle of the delta trap, and it is important not to place any stickers over this hole so water can properly drain from the trap.


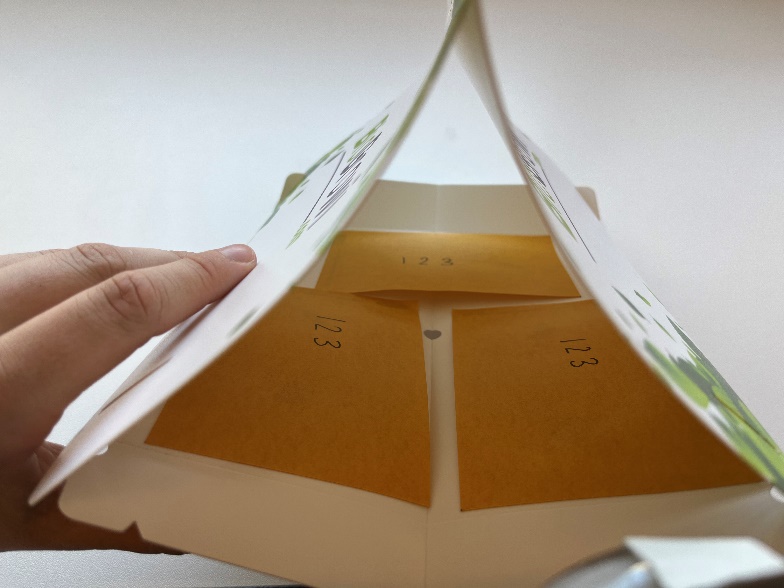


1. Wash your hands with soap before proceeding.
2. Remove the yellow protective layer. It’s easiest to start from the back, so you can avoid touching the exposed stickers with your hands. Avoid pulling yellow protective layer upwards as much as possible, but peel it off with a more horizontal motion. To keep the sticky seal in place, you can hold down the edge of the transparent seal (with the Bio-Rad logo) with your finger. **It is very important to keep the yellow protective layer, as it is essential for returning the stickers.** Store the yellow protective layer in the provided resealable plastic zip-lock bag.
3. Fold the other side of the delta trap upwards. Like with the first side, the corners should fit perfectly into the slits of the trap’s "roof."
4. Now choose a good location to hang the trap. The location should meet the following criteria:
   - The trap is hung outside.
   - The trap can be hung securely.
   - The trap is out of reach of small children and pets.
   - The trap can hang undisturbed for 4 weeks.

Good examples include hanging it from a tree branch, the railing of a balcony, or a laundry line.

1. Hang the delta trap in the chosen spot by threading the piece of rope through two of the holes at the top of the trap. Tie a knot to securely hang the trap in your chosen position.
2. Let the delta trap hang for 4 weeks.

**Collecting the trap and returning the sticky seals (After 4 weeks)**

1. Untie the string and take the trap down from the chosen location.
2. Wash your hands with soap before proceeding.
3. Open the trap on the side of the horizontal sticker. Carefully remove the stickers from the trap, try to touch only the non-sticky part/strip (with Bio-Rad logo) of the stickers.
4. On the yellow protective layer you removed before, there is a number indicating the top. Reapply the yellow protective layer to the stickers. **Make sure to stick the unmarked side of the yellow protective layer onto the sticky side of the sticker.** If this is done incorrectly, with the marked side of the cover stuck to the seal, the cover can no longer be removes and the measurement will be unusable for us.
5. Remove the adhesive putties from the stickers.
6. Fill out the small included information sheet. Then place the sheet and the three stickers back in the resealable plastic bag. Place this bag in the return envelope.
7. If you cannot not send the return envelope immediately, store it in a dry and dark place at room temperature.
8. The return envelope can be posted in a mailbox without a stamp (free of charge).
